# Supplementary material for: Patient-Reported Financial Distress in Cancer: A Systematic Review of Risk Factors in Universal Healthcare Systems
Source: Cancers (Basel). 2021 Oct 7;13(19):5015. doi: 10.3390/cancers13195015 (PMC8508394; doi:10.3390/cancers13195015)
Supplement: Supplementary file 1 [file cancers-13-05015-s001.zip › cancers-1281264-supplementary.pdf]

Supplementary material

Supplementary material 1: Search strategy

Search strategy for Pubmed performed up to December 2020

((Cancer*[Title/Abstract]) OR (Tumor*[Title/Abstract]) OR (Oncolog*[Title/Abstract]) OR (Neoplasm*[Title/Abstract]) OR (Carcinoma*[Title/Abstract]))

AND

(("Financial distress"[Title/Abstract]) OR ("Financial difficult*"[Title/Abstract]) OR ("Financial consequence*"[Title/Abstract]) OR ("Financial toxicit*"[Title/Abstract]) OR ("Financial hardship*"[Title/Abstract]) OR ("Financial stress"[Title/Abstract]) OR ("Financial burden"[Title/Abstract]) OR ("Financial strain"[Title/Abstract]) OR ("Financial worr*"[Title/Abstract]) OR ("Financial catastrophe*"[Title/Abstract]) OR ("Financial constraint*"[Title/Abstract]) OR ("Financial well-being"[Title/Abstract]) OR ("Financial support"[Title/Abstract]) OR (Bankrupt*[Title/Abstract]) OR (Insolvency[Title/Abstract]) OR ("Financial problem*"[Title/Abstract]) OR ("Financial effect*"[Title/Abstract]) OR ("Financial issue*"[Title/Abstract]))

Filter: English, German

Search strategy was adjusted to PsycInfo and CINAHL including similar search terms and restrictions.

Supplementary material 2: Study characteristics

Table S2: Study characteristics

Study	Study Design	Country	Time point of measurement	Sample size	Cancer entity	Age	Gender
Quantitative studies							
Akechi et al. (2012) [1]	Cross sectional study	Japan	Any time point after diagnosis	N= 619	• Gynaecological<br>• Gastrointestinal<br>• Haematological malignancies<br>• Thorac<br>• Other	<70: 506, >70: 113	Female: 536, Male: 83
Arndt et al. (2004) [2]	Cross sectional study	Germany	1 year after diagnosis	N= 309	• Gastrointestinal	Mean: 65.1 (18-80)	Female: 135, Male: 174
Arndt et al. (2017) [3]	Cross sectional study	Germany	Cancer during 1994–2004, survived cancer at least 5 years were recruited in 2009–2011	N= 8830	• Gynaecological<br>• Gastrointestinal<br>• Urogenital	Mean: 67.3	Female: 4294; Male: 4536
Arndt et al. (2019) [4]	Cross sectional study	Germany	Cancer during 1994–2004, survived cancer at least 5 years were recruited in 2009–2011	N= 2163	• Gynaecological<br>• Gastrointestinal<br>• Urogenital	Mean: 50.1 (18-59)	Female: 1191, Male:972
Barbaret et al. (2017) [5]	Cross sectional study	France	Outpatients or hospitalized patients	N= 143	• Gynaecological<br>• Gastrointestinal<br>• Thorac<br>• Urogenital	Mean: 58	Female: 67, Male: 76
Barbaret et al. (2019) [6]	Cross sectional study	France & USA	Outpatients or hospitalized patients	N= 143	• Gynaecological<br>• Gastrointestinal<br>• Thorac<br>• Urogenital	Mean: 58	Female: 70, Male: 73
Büttner et al. (2019) [7]	Cohort study	Germany	At the end of their hospital stay (t0), with follow up 3 (t1), and 15 (t2) months after t0	N= 502	• Gynaecological<br>• Gastrointestinal<br>• Head and Neck<br>• Urogenital<br>• Other	Mean: 45.9 (19-57)	Female: 234, Male: 268
Doege et al. (2019) [8]	Cross sectional study	Germany	Long-term cancer survivors diagnosed between 1994 and 2004	N= 3652	• Gynaecological	Mean: 63.6	Female only
Egestad and Nieder (2015) [9]	Cohort study	Norway	At baseline: first week of radiation treatment, and during the last week after administration of 60 Gy.	N= 67	• Head and Neck	Mean: 60	Female: 18, Male: 49

Ezeife et al. (2018) [9,10]	Cross sectional study	Canada	Diagnosis at least 3 months + receiving treatment	N= 200	• Thorac	32-86	Female: 111, Male: 88
Gordon et al. (2017a) [11]	Cross sectional study	Australia	No restrictions	N= 289	• Urogenital	Mean: 65.1	Men only
Gordon et al. (2017b) [12]	Cohort study	Australia	6 months (T0) and 12 months (T1) post-colorectal cancer diagnosis	N= 187	• Gastrointestinal	Mean: 56.3 (45-64)	Female: 64, Male: 123
Gordon et al. (2020) [13]	Cross sectional study	Australia	No restrictions	N= 204	• Gastrointestinal	Mean: 58.7 (18 -83)	Female: 102, Male: 102
Hanly et al. (2018) [14]	Cross sectional study	Ireland	Diagnosed October 2007 to September 2009 and still alive in January 2010	N= 496	• Gastrointestinal	<65: 199, 65-74: 167, 75+: 130	Female: 186, Male: 310
Honda et al. (2019) [15]	Cross sectional study	Japan	Patients receiving anticancer drug therapy	N= 156	• Gastrointestinal<br>• Head and Neck<br>• Other	Mean: 67 (30-87)	Female: 73, Male: 83
Ivanauskienė et al. (2014) [16]	Cross sectional study	Lithuania	Diagnosis registered between January 1, 2011 and December 31, 2011	N= 338	• Gynaecological	Mean: 58.6 (28–95)	Female only
Kaptein et al. (2013) [17]	Cohort study	Japan & Netherlands	Before their first chemotherapy course (t0), one week after their first chemotherapy course (t1), and 8 weeks after the start of chemotherapy (t2)	N= 43	• Gynaecological	Mean: 48.3 (35-68)	Not reported
Koch et al. (2013) [18]	Cohort study	Germany	Baseline (a few days after diagnosis) and four follow-up time-points after one (FU1), three (FU3), five to seven (FU5), and 10 to 12 (FU10) years post-diagnosis.	N= 387	• Gynaecological	Mean t0: 57,9; Mean t10: 55, 6 (18-80)	Female only
Koskinen et al. (2019) [19]	Cross sectional study	Finland	All disease states from diagnosis to end-of-life care	N= 1978	• Gynaecological<br>• Gastrointestinal<br>• Urogenital	Median: 66	Female: 905, Male: 1073
Lu et al. (2019) [20]	Cross sectional study	Ireland	At least 8 months post diagnosis	N= 531	• Head and Neck	<50: 142, 50-64: 254, >64: 133	Female: 170, Male: 361
Mandaliya et al. (2016) [21]	Cross sectional study	Australia	3 to 5 years since last treatment	N= 45	• Gynaecological<br>• Gastrointestinal<br>• Thorac<br>• Other	Not reported	Female: 29, Male: 16
McLean et al. (2020) [22]	Cross sectional study	Australia	Patients actively receiving systemic therapy	N= 53	• Other	Median: 63.5	Female: 29, Male: 24

McNeil et al. (2019) [23]	Cross sectional study	Australia	Six to 24 months from diagnosis	N= 196	• Haematological malignancies<br>• Head and Neck<br>• Skin<br>• Urogenital<br>• Other	Mean: 21.6 (15-27)	Female: 97, Male: 99
Mehlis et al. (2020) [24]	Cross sectional study	Germany	Cancer patients with ongoing disease	N= 247	• Gastrointestinal	Median: 63	Female: 88; Male: 159
Mercadante et al. (2020) [25]	Cross sectional study	Italy	Not reported	N= 236	• Gynaecological<br>• Gastrointestinal<br>• Head and Neck<br>• Thorac<br>• Urogenital<br>• Other	Mean: 63.9	Female: 122, Male: 114
Paul et al. (2013) [26]	Cross sectional study	Australia	Diagnosed in the previous 3 years	N= 268	• Haematological malignancies	Mean: 59.5	Female: 111, Male: 157
Pearce et al. (2019) [27]	Cross sectional study	Netherlands	Cancer survivors who participated in cross-sectional and longitudinal studies conducted between 2004 and 2015	N= 2931	• Haematological malignancies<br>• Gynaecological<br>• Gastrointestinal<br>• Head and Neck<br>• Skin<br>• Urogenital	Mean: 55.3	Female: 1471, Male: 1460
Schmidt et al. (2019) [28]	Cohort study	Germany	Newly diagnosed patients between 2010 and 2013 (t0), 5 year after surgery (t1)	N= 137	• Gynaecological<br>• Urogenital	Mean: 50.5	Female only
Sharp and Timmons (2016) [29]	Cross sectional study	Ireland	Diagnosed with cancer 3–24 months previously	N= 698	• Gynaecological<br>• Urogenital	Mean: 57.5	Female: 486, Male: 212
Zucca et al. (2011) [30]	Cohort study	Australia	6 and 12 months after diagnosis	N= 1410	• Haematological malignancies<br>• Gynaecological<br>• Gastrointestinal<br>• Head and Neck<br>• Skin<br>• Thorac<br>• Urogenital	18-80	Female: 567, Male: 843
Qualitative studies							
Amir et al. (2012) [31]	Cross sectional study	England	At least 16 weeks post-diagnosis	N= 40	• Unspecified	20-89	Female: 21, Male: 19
Bennett et al. (2009) [32]	Cross sectional study	New Zealand	6–24 months since diagnosis	N= 68	• Unspecified	Men 52.3 (22–74)	Female: 47, Male: 21
Fitch and Longo (2018) [33]	Cross sectional study	Canada	Receiving active treatment for at least a month	N= 14	• Gynaecological<br>• Gastrointestinal<br>• Thorac<br>• Urogenital	Average: 51.7 (37-78)	Not reported

Fitch et al. (2020) [34]	Cross sectional study	Canada	Minimum of 4 weeks of cancer treatment still on active treatment +a subset of patients beyond active treatment	N= 378	• Gynaecological<br>• Gastrointestinal<br>• Thorac<br>• Urogenital	18-24: 20; 35-54: 102; 55+: 256	Female: 227, Male: 151
Gray et al. (2004) [35]	Cross sectional study	Canada	No restrictions	N= 276	• Gynaecological	<50 years: 78; 50+ years: 199	Female only
Grinyer (2007) [36]	Cross sectional study	England	Between the ages of 15 and 25 years when diagnosed with cancer	N= 20	• Unspecified	16-26	Not reported
Kane et al. (2020) [37]	Cross sectional study	Canada	No restrictions	N= 8	• Gynaecologica<br>• Haematological malignancies	45-62	Female: 7, male: 1
McGrath (1999) [38]	Cross sectional study	Australia	No restrictions	N= 18	• Gynaecological	35-49: 33%, 50-64: 50%, >65: 17%	Female only
McGrath (2000) [39]	Cross sectional study	Australia	Not stated	N= 10	• Haematological malignancies	17-53	Female: 8, Male: 2
Moffatt et al. (2010) [40]	Cross sectional study	England	Patients between June 2008 and March 2009 using welfare rights service	N= 22	• Unspecified	35-83	Female: 11, Male: 11
Moffatt et al. (2012) [41]	Cross sectional study	England	Cancer patients who accessed wellfare service between April 2009 and March 2010.	N= 36	• Unspecified	Median: 62	Female: 17, Male: 19
Moffatt and Noble (2015) [42]	Cohort study	England	Cancer patients who accessed wellfare service between April 2009 and March 2010.	N= 23	• Unspecified	32-62	
Schröder et al. (2020) [43]	Cross sectional study	Germany	Had completed acute treat- ment for cancer within the last 5 years	N= 39	• Gynaecological<br>• Gastrointestinal<br>• Thorac<br>• Urogenital	40-86	Female: 18, Male: 21
Slavova-Azmanova et al. (2020) [44]	Cross sectional study	Australia	Participated in a cross-sectional survey between 2014 and 2017	N= 300	• Gynaecological<br>• Gastrointestinal<br>• Thorac<br>• Urogenital	Mean: 64	Female: 155, Male: 145
Timmons et al. (2013a) [45]	Cross sectional study	Ireland	Completed initial treatment	N= 20	• Gynaecological<br>• Thorac<br>• Urogenital	>50: 7, 50-59: 8, 60-69: 4, >70: 1	Female: 14, Male: 6
Timmons et al. (2013b) [46]	Cross sectional study	Ireland	Post- initial treatment	N= 20	• Gynaecological<br>• Thorac<br>• Urogenital	>50: 7, 50-59: 8, 60-69: 4, >70: 1	Female: 14, Male: 6

References

1. Akechi, T.; Okuyama, T.; Uchida, M.; Nakaguchi, T.; Ito, Y.; Yamashita, H.; Toyama, T.; Komatsu, H.; Kizawa, Y.; Wada, M. Perceived needs, psychological distress and quality of life of elderly cancer patients. *Jpn. J. Clin. Oncol.* **2012**, *42*, 704–710, doi:10.1093/jjco/hys075.
2. Arndt, V.; Merx, H.; Stegmaier, C.; Ziegler, H.; Brenner, H. Quality of life in patients with colorectal cancer 1 year after diagnosis compared with the general population: a population-based study. *J. Clin. Oncol.* **2004**, *22*, 4829–4836, doi:10.1200/JCO.2004.02.018.
3. Arndt, V.; Koch-Gallenkamp, L.; Jansen, L.; Bertram, H.; Eberle, A.; Holleczek, B.; Schmid-Höpfner, S.; Waldmann, A.; Zeissig, S.R.; Brenner, H. Quality of life in long-term and very long-term cancer survivors versus population controls in Germany. *Acta Oncol.* **2017**, *56*, 190–197, doi:10.1080/0284186X.2016.1266089.
4. Arndt, V.; Koch-Gallenkamp, L.; Bertram, H.; Eberle, A.; Holleczek, B.; Pritzkuleit, R.; Waldeyer-Sauerland, M.; Waldmann, A.; Zeissig, S.R.; Doege, D.; et al. Return to work after cancer. A multi-regional population-based study from Germany. *Acta Oncol.* **2019**, *58*, 811–818, doi:10.1080/0284186X.2018.1557341.
5. Barbaret, C.; Brosse, C.; Rhondali, W.; Ruer, M.; Monsarrat, L.; Michaud, P.; Schott, A.M.; Delgado-Guay, M.; Bruera, E.; Sanchez, S.; et al. Financial distress in patients with advanced cancer. *PLoS One* **2017**, *12*, e0176470, doi:10.1371/journal.pone.0176470.
6. Barbaret, C.; Delgado-Guay, M.O.; Sanchez, S.; Brosse, C.; Ruer, M.; Rhondali, W.; Monsarrat, L.; Michaud, P.; Schott, A.M.; Bruera, E.; et al. Inequalities in Financial Distress, Symptoms, and Quality of Life Among Patients with Advanced Cancer in France and the U.S. *Oncologist* **2019**, *24*, 1121–1127, doi:10.1634/theoncologist.2018-0353.
7. Büttner, M.; König, H.-H.; Löbner, M.; Briest, S.; Konnopka, A.; Dietz, A.; Riedel-Heller, S.; Singer, S. Out-of-pocket-payments and the financial burden of 502 cancer patients of working age in Germany: results from a longitudinal study. *Support. Care Cancer* **2019**, *27*, 2221–2228, doi:10.1007/s00520-018-4498-1.
8. Doege, D.; Thong, M.S.-Y.; Koch-Gallenkamp, L.; Bertram, H.; Eberle, A.; Holleczek, B.; Pritzkuleit, R.; Waldeyer-Sauerland, M.; Waldmann, A.; Zeissig, S.R.; et al. Health-related quality of life in long-term disease-free breast cancer survivors versus female population controls in Germany. *Breast Cancer Res. Treat.* **2019**, *175*, 499–510, doi:10.1007/s10549-019-05188-x.
9. Egestad, H.; Nieder, C. Undesirable financial effects of head and neck cancer radiotherapy during the initial treatment period. *Int. J. Circumpolar Health* **2015**, *74*, 26686, doi:10.3402/ijch.v74.26686.
10. Ezeife, D.A.; Morganstein, B.J.; Lau, S.; Law, J.H.; Le, L.W.; Bredle, J.; Cella, D.; Doherty, M.K.; Bradbury, P.; Liu, G.; et al. Financial Burden Among Patients With Lung Cancer in a Publically Funded Health Care System. *Clin. Lung Cancer* **2019**, *20*, 231–236, doi:10.1016/j.cllc.2018.12.010.
11. Gordon, L.G.; Walker, S.M.; Mervin, M.C.; Lowe, A.; Smith, D.P.; Gardiner, R.A.; Chambers, S.K. Financial toxicity: a potential side effect of prostate cancer treatment among Australian men. *Eur. J. Cancer Care (Engl)* **2017**, *26*, doi:10.1111/ecc.12392.
12. Gordon, L.G.; Beesley, V.L.; Mihala, G.; Koczwara, B.; Lynch, B.M. Reduced employment and financial hardship among middle-aged individuals with colorectal cancer. *Eur. J. Cancer Care (Engl)* **2017**, *26*, doi:10.1111/ecc.12744.
13. Gordon, L.G.; Elliott, T.M.; Wakelin, K.; Leyden, S.; Leyden, J.; Michael, M.; Pavlakis, N.; Mumford, J.; Segelov, E.; Wyld, D.K. The Economic Impact on Australian Patients with Neuroendocrine Tumours. *Patient* **2020**, *13*, 363–373, doi:10.1007/s40271-020-00412-z.
14. Hanly, P.; Maguire, R.; Ceilleachair, A.O.; Sharp, L. Financial hardship associated with colorectal cancer survivorship: The role of asset depletion and debt accumulation. *Psychooncology.* **2018**, *27*, 2165–2171, doi:10.1002/pon.4786.
15. Honda, K.; Gyawali, B.; Ando, M.; Kumanishi, R.; Kato, K.; Sugiyama, K.; Mitani, S.; Masuishi, T.; Narita, Y.; Bando, H.; et al. Prospective Survey of Financial Toxicity Measured by the Comprehensive Score for Financial Toxicity in Japanese Patients With Cancer. *J. Glob. Oncol.* **2019**, *5*, 1–8, doi:10.1200/JGO.19.00003.
16. Ivanauskienė, R.; Padaiga, Ž.; Šimoliūnienė, R.; Smailytė, G.; Domeikienė, A. Well-being of newly diagnosed women with breast cancer: which factors matter more? *Support. Care Cancer* **2014**, *22*, 519–526, doi:10.1007/s00520-013-2005-2.
17. Kaptein, A.A.; Yamaoka, K.; Snoei, L.; van der Kloot, W.A.; Inoue, K.; Tabei, T.; Kroep, J.R.; Krol-Warmerdam, E.; Ranke, G.; Meirink, C.; et al. Illness perceptions and quality of life in Japanese and Dutch women with breast cancer. *J. Psychosoc. Oncol.* **2013**, *31*, 83–102, doi:10.1080/07347332.2012.741092.
18. Koch, L.; Jansen, L.; Herrmann, A.; Stegmaier, C.; Holleczek, B.; Singer, S.; Brenner, H.; Arndt, V. Quality of life in long-term breast cancer survivors - a 10-year longitudinal population-based study. *Acta Oncol.* **2013**, *52*, 1119–1128, doi:10.3109/0284186X.2013.774461.
19. Koskinen, J.-P.; Färkkilä, N.; Sintonen, H.; Saarto, T.; Taari, K.; Roine, R.P. The association of financial difficulties and out-of-pocket payments with health-related quality of life among breast, prostate and colorectal cancer patients. *Acta Oncol.* **2019**, *58*, 1062–1068, doi:10.1080/0284186X.2019.1592218.
20. Lu, L.; O'Sullivan, E.; Sharp, L. Cancer-related financial hardship among head and neck cancer survivors: Risk factors and associations with health-related quality of life. *Psychooncology.* **2019**, *28*, 863–871, doi:10.1002/pon.5034.
21. Mandaliya, H.; Ansari, Z.; Evans, T.; Oldmeadow, C.; George, M. Psychosocial Analysis of Cancer Survivors in Rural Australia: Focus on Demographics, Quality of Life and Financial Domains. *Asian Pac. J. Cancer Prev.* **2016**, *17*, 2459–2464.
22. McLean, L.; Hong, W.; McLachlan, S.-A. Financial toxicity in patients with cancer attending a public Australian tertiary hospital: A pilot study. *Asia Pac. J. Clin. Oncol.* **2020**, doi:10.1111/ajco.13448.
23. McNeil, R.J.; McCarthy, M.; Dunt, D.; Thompson, K.; Kosola, S.; Orme, L.; Drew, S.; Sawyer, S. Financial Challenges of Cancer for Adolescents and Young Adults and Their Parent Caregivers. *Social Work Research* **2019**, *43*, 17–30, doi:10.1093/swr/svy027.
24. Mehlis, K.; Witte, J.; Surmann, B.; Kudlich, M.; Apostolidis, L.; Walther, J.; Jäger, D.; Greiner, W.; Winkler, E.C. The patient-level effect of the cost of Cancer care - financial burden in German Cancer patients. *BMC Cancer* **2020**, *20*, 529, doi:10.1186/s12885-020-07028-4.
25. Mercadante, S.; Aielli, F.; Adile, C.; Bonanno, G.; Casuccio, A. Financial distress and its impact on symptom expression in advanced cancer patients. *Support. Care Cancer* **2021**, *29*, 485–490, doi:10.1007/s00520-020-05507-9.

26. Paul, C.L.; Hall, A.E.; Carey, M.L.; Cameron, E.C.; Clinton-McHarg, T. Access to care and impacts of cancer on daily life: do they differ for metropolitan versus regional hematological cancer survivors? *J. Rural Health* **2013**, *29 Suppl 1*, s43-50, doi:10.1111/jrh.12020.
27. Pearce, A.; Tomalin, B.; Kaambwa, B.; Horevoorts, N.; Duijts, S.; Mols, F.; van de Poll-Franse, L.; Koczwara, B. Financial toxicity is more than costs of care: the relationship between employment and financial toxicity in long-term cancer survivors. *J. Cancer Surviv.* **2019**, *13*, 10–20, doi:10.1007/s11764-018-0723-7.
28. Schmidt, M.E.; Scherer, S.; Wiskemann, J.; Steindorf, K. Return to work after breast cancer: The role of treatment-related side effects and potential impact on quality of life. *Eur. J. Cancer Care (Engl)* **2019**, *28*, e13051, doi:10.1111/ecc.13051.
29. Sharp, L.; Timmons, A. Pre-diagnosis employment status and financial circumstances predict cancer-related financial stress and strain among breast and prostate cancer survivors. *Support. Care Cancer* **2016**, *24*, 699–709, doi:10.1007/s00520-015-2832-4.
30. Zucca, A.; Boyes, A.; Newling, G.; Hall, A.; Girgis, A. Travelling all over the countryside: travel-related burden and financial difficulties reported by cancer patients in New South Wales and Victoria. *Aust. J. Rural Health* **2011**, *19*, 298–305, doi:10.1111/j.1440-1584.2011.01232.x.
31. Amir, Z.; Wilson, K.; Hennings, J.; Young, A. The meaning of cancer: implications for family finances and consequent impact on lifestyle, activities, roles and relationships. *Psychooncology.* **2012**, *21*, 1167–1174, doi:10.1002/pon.2021.
32. Bennett, J.A.; Brown, P.; Cameron, L.; Whitehead, L.C.; Porter, D.; McPherson, K.M. Changes in employment and household income during the 24 months following a cancer diagnosis. *Support. Care Cancer* **2009**, *17*, 1057–1064, doi:10.1007/s00520-008-0540-z.
33. Fitch, M.; Longo, C.J. Exploring the impact of out-of-pocket costs on the quality of life of Canadian cancer patients. *J. Psychosoc. Oncol.* **2018**, *36*, 582–596, doi:10.1080/07347332.2018.1486937.
34. Fitch, M.I.; Longo, C.J.; Chan, R.J. Cancer patients' perspectives on financial burden in a universal healthcare system: Analysis of qualitative data from participants from 20 provincial cancer centers in Canada. *Patient Educ. Couns.* **2021**, *104*, 903–910, doi:10.1016/j.pec.2020.08.013.
35. Gray, R.E.; James, P.; Manthorne, J.; Gould, J.; Fitch, M.I. A consultation with Canadian rural women with breast cancer. *Health Expect.* **2004**, *7*, 40–50, doi:10.1046/j.1369-6513.2003.00248.x.
36. Grinyer, A. The biographical impact of teenage and adolescent cancer. *Chronic Illn.* **2007**, *3*, 265–277, doi:10.1177/1742395307085335.
37. Kane, D.; Rajacich, D.; Andary, C. Experiences of cancer patients' return to work. *Can. Oncol. Nurs. J.* **2020**, *30*, 113–118, doi:10.5737/23688076302113118.
38. McGrath, P.; Patterson, C.; Yates, P.; Treloar, S.; Oldenburg, B.; Loos, C. A study of postdiagnosis breast cancer concerns for women living in rural and remote Queensland. Part II: Support issues. *Aust. J. Rural Health* **1999**, *7*, 43–52, doi:10.1046/j.1440-1584.1999.00216.x.
39. McGrath, P. "It's horrendous--but really, what can you do?" Preliminary findings on financial impact of relocation for specialist treatment. *Aust. Health Rev.* **2000**, *23*, 94–103, doi:10.1071/ah000094.
40. Moffatt, S.; Noble, E.; Exley, C. "Done more for me in a fortnight than anybody done in all me life." How welfare rights advice can help people with cancer. *BMC Health Serv. Res.* **2010**, *10*, 259, doi:10.1186/1472-6963-10-259.
41. Moffatt, S.; Noble, E.; White, M. Addressing the financial consequences of cancer: qualitative evaluation of a welfare rights advice service. *PLoS One* **2012**, *7*, e42979, doi:10.1371/journal.pone.0042979.
42. Moffatt, S.; Noble, E. Work or welfare after cancer? Explorations of identity and stigma. *Sociol. Health Illn.* **2015**, *37*, 1191–1205, doi:10.1111/1467-9566.12303.
43. Schröder, S.L.; Schumann, N.; Fink, A.; Richter, M. Coping mechanisms for financial toxicity: a qualitative study of cancer patients' experiences in Germany. *Support. Care Cancer* **2020**, *28*, 1131–1139, doi:10.1007/s00520-019-04915-w.
44. Slavova-Azmanova, N.S.; Newton, J.C.; Saunders, C.; Johnson, C.E. 'Biggest factors in having cancer were costs and no entitlement to compensation'-The determinants of out-of-pocket costs for cancer care through the lenses of rural and outer metropolitan Western Australians. *Aust. J. Rural Health* **2020**, *28*, 588–602, doi:10.1111/ajr.12686.
45. Timmons, A.; Gooberman-Hill, R.; Sharp, L. "It's at a time in your life when you are most vulnerable": a qualitative exploration of the financial impact of a cancer diagnosis and implications for financial protection in health. *PLoS One* **2013**, *8*, e77549, doi:10.1371/journal.pone.0077549.
46. Timmons, A.; Gooberman-Hill, R.; Sharp, L. The multidimensional nature of the financial and economic burden of a cancer diagnosis on patients and their families: qualitative findings from a country with a mixed public-private healthcare system. *Support. Care Cancer* **2013**, *21*, 107–117, doi:10.1007/s00520-012-1498-4.

Supplementary material 3: Healthcare expenditure by type of financing

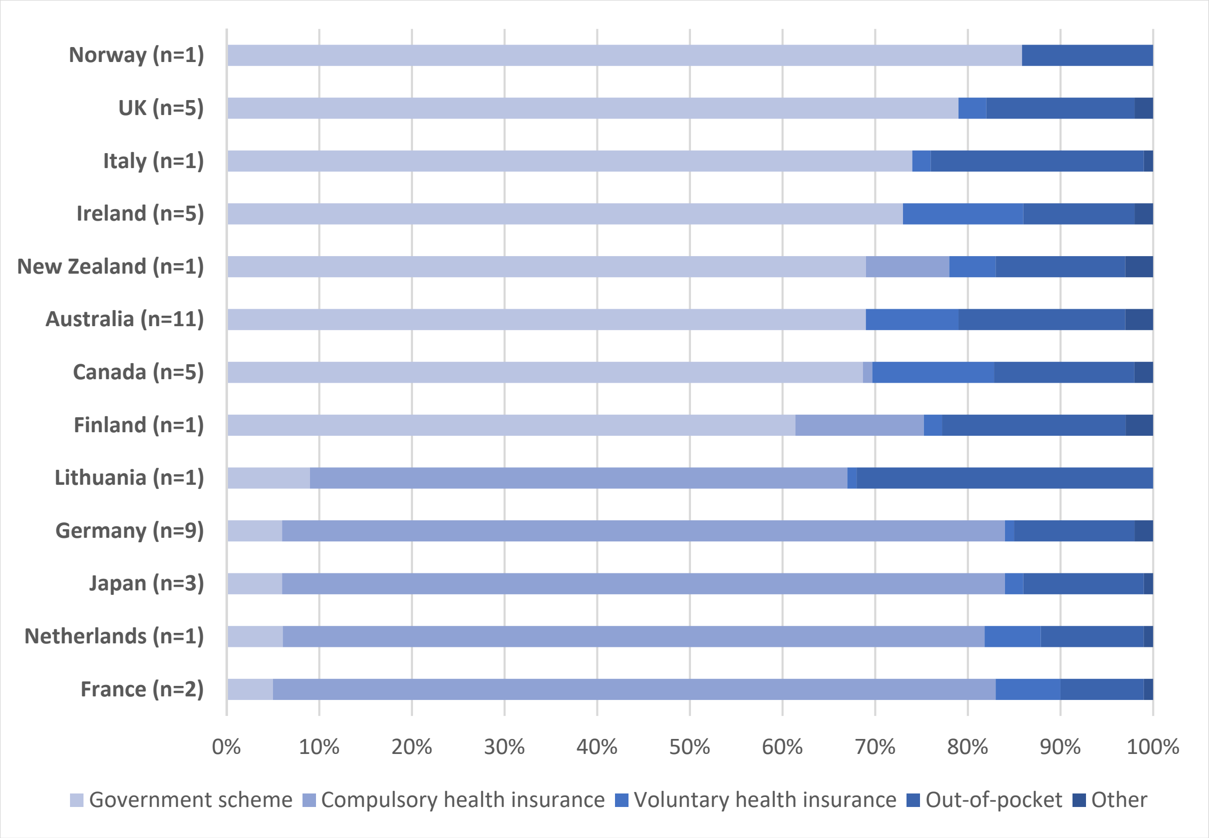

Figure S3: Health expenditure by type of financing; data based on OECD data from 2017 (or nearest year available) [47]

References

1. OECD. *Health at a Glance 2019*; OECD, 2019, ISBN 9789264382084.

Supplementary material 4: Quality appraisal

Quality appraisal of quantitative studies based on NIH Quality Assessment Tool for Observational Cohort and Cross-Sectional Studies [48]

Table S4.1: Quality appraisal of quantitative studies

Study	Was the research question or objective in this paper clearly stated?	Was the study population clearly specified and defined?	Was the participation rate of eligible persons at least 50%?	Were all the subjects selected or recruited from the same or similar populations (including the same time period)? Were inclusion and exclusion criteria for being in the study prespecified and applied uniformly to all participants?	Was a sample size justification, power description, or variance and effect estimates provided?	For the analyses in this paper, were the exposure(s) of interest measured prior to the outcome(s) being measured?	Was the timeframe sufficient so that one could reasonably expect to see an association between exposure and outcome if it existed?	For exposures that can vary in amount or level, did the study examine different levels of the exposure as related to the outcome (e.g., categories of exposure, or exposure measured as continuous variable)?	Were the exposure measures (independent variables) clearly defined, valid, reliable, and implemented consistently across all study participants?	Was the exposure(s) assessed more than once over time?	Were the outcome measures (dependent variables) clearly defined, valid, reliable, and implemented consistently across all study participants?	Were the outcome assessors blinded to the exposure status of participants?	Was loss to follow-up after baseline 20% or less?	Were key potential confounding variables measured and adjusted statistically for their impact on the relationship between exposure(s) and outcome(s)?	Quality rating (good, fair or poor)
Akechi et al. (2012)[1]	Yes	Yes	Yes	No	No	No	No	Yes	Yes	NA	Yes	NA	NA	Yes	Fair
Arndt et al. (2004)	Yes	Yes	Yes	Yes	No	No	No	Yes	Yes	NA	Yes	NA	NA	Yes	Fair
Arndt et al. (2017)[3]	Yes	Yes	No	No	No	No	No	Yes	Yes	NA	Yes	NA	NA	Yes	Fair
Arndt et al. (2019)[4]	Yes	Yes	No	No	No	No	No	Yes	Yes	NA	Yes	NA	NA	Yes	Fair
Barbaret et al. (2017)[5]	Yes	Yes	NR	Yes	No	No	No	Yes	No	NA	No	NA	NA	No	Poor
Barbaret et al. (2019)[6]	Yes	Yes	NR	Yes	No	No	No	Yes	No	NA	No	NA	NA	Yes	Fair

Büttner et al. (2019)[7]	Yes	Yes	NR	Yes	No	Yes	Yes	Yes	Yes	Yes	Yes	NA	No	No	Fair
Doege et al. (2019)[8]	Yes	Yes	No	No	No	No	No	Yes	Yes	NA	Yes	NA	NA	Yes	Fair
Egestad and Nieder (2015)[9]	Yes	Yes	NR	Yes	No	Yes	Yes	Yes	Yes	Yes	Yes	NA	Yes	No	Fair
Ezeife et al. (2018)[10]	Yes	No	Yes	Yes	No	No	No	Yes	Yes	NA	Yes	NA	NA	Yes	Fair
Gordon et al. (2017a)[11]	Yes	Yes	NR	Yes	Yes	No	No	Yes	Yes	NA	No	NA	NA	No	Fair
Gordon et al. (2017b)[12]	Yes	Yes	No	Yes	No	Yes	Yes	Yes	Yes	Yes	Yes	NA	Yes	Yes	Fair
Gordon et al. (2020)[13]	Yes	Yes	No	Yes	No	No	No	Yes	Yes	NA	Yes	NA	NA	No	Fair
Hanly et al. (2018)[14]	Yes	Yes	No	Yes	No	No	No	Yes	Yes	NA	No	NA	NA	Yes	Fair
Honda et al. (2019)[15]	Yes	Yes	Yes	Yes	No	No	No	Yes	Yes	NA	Yes	NA	NA	Yes	Fair
Ivanauskienė et al. (2014)[16]	Yes	Yes	Yes	Yes	Yes	No	No	Yes	Yes	NA	Yes	NA	NA	Yes	Good
Kaptein et al. (2013)[17]	Yes	Yes	NR	Yes	No	Yes	No	Yes	Yes	Yes	Yes	NA	NR	Yes	Fair
Koch et al. (2013)[18]	Yes	Yes	Yes	Yes	No	Yes	Yes	Yes	Yes	Yes	Yes	NA	No	Yes	Fair
Koskinen et al. (2019)[19]	Yes	Yes	NR	Yes	No	No	No	Yes	Yes	NA	Yes	NA	NA	Yes	Fair
Lu et al. (2019)[20]	Yes	Yes	Yes	Yes	No	No	No	Yes	Yes	NA	No	NA	NA	Yes	Fair
Mandaliya et al. (2016)[21]	Yes	Yes	NR	Yes	No	No	No	Yes	Yes	NA	Yes	NA	NA	Yes	Fair
McLean et al. (2020)[22]	Yes	Yes	Yes	Yes	No	No	No	Yes	Yes	NA	Yes	NA	NA	Yes	Fair
McNeil et al. (2019)[23]	Yes	Yes	No	Yes	No	No	No	Yes	Yes	NA	No	NA	NA	Yes	Fair
Mehlis et al. (2020)[24]	Yes	Yes	Yes	Yes	No	No	No	Yes	Yes	NA	No	NA	NA	Yes	Fair
Mercadante et al. (2020)[25]	Yes	Yes	NR	Yes	No	No	No	Yes	Yes	NA	No	NA	NA	Yes	Fair
Paul et al. (2013)[26]	Yes	Yes	No	Yes	No	No	No	Yes	Yes	NA	No	NA	NA	No	Fair
Pearce et al. (2019)[27]	Yes	Yes	NR	Yes	No	No	No	Yes	Yes	NA	Yes	NA	NA	Yes	Fair
Schmidt et al. (2019)[28]	Yes	Yes	NR	No	No	Yes	Yes	Yes	Yes	Yes	Yes	NA	NR	Yes	Fair

Sharp and Timmons (2016)[29]	Yes	Yes	Yes	Yes	No	No	No	Yes	Yes	NA	No	NA	NA	Yes	Fair
Zucca et al. (2011)[30]	Yes	Yes	Yes	Yes	No	Yes	Yes	Yes	Yes	Yes	Yes	NA	NR	Yes	Fair

Qualitative appraisal of qualitative studies based on CASP Checklist ***[49]***

Table S4.2: Quality appraisal of qualitative studies

**Study**	**Was there a clear statement of the aims of the research??**	**Is a qualitative methodology appropriate?**	**Was the research design appropriate to address the aims of the research?**	**Was the recruitment strategy appropriate to the aims of the research?**	**Was the data collected in a way that addressed the research issue?**	**Has the relationship between researcher and participants been adequately considered?**	**Have ethical issues been taken into consideration?**	**Was the data analysis sufficiently rigorous?**	**Is there a clear statement of findings?**	**How valuable is the research?**
Amir et al. (2012)[31]	Yes	Yes	Yes	Yes	Yes	No	Yes	Yes	Yes	Yes
Bennett et al. (2009)[32]	Yes	Yes	Yes	Yes	Yes	No	Yes	No	Yes	Yes
Fitch and Longo (2018) [33]	Yes	Yes	Yes	Yes	Yes	No	Yes	Yes	Yes	Yes
Fitch et al. (2020) [34]	Yes	Yes	Yes	NA	Yes	NA	No	Yes	Yes	Yes
Gray et al. (2004) [35]	Yes	Yes	Yes	Yes	Yes	No	No	Yes	Yes	Yes
Grinyer (2007) [36]	Yes	Yes	Yes	Yes	Yes	No	Yes	Yes	Yes	Yes
Kane et al. (2020) [37]	Yes	Yes	Yes	Yes	Yes	No	Yes	Yes	Yes	Yes
McGrath (1999) [38]	Yes	Yes	Yes	Yes	Yes	No	No	No	Yes	No
McGrath (2000) [39]	Yes	Yes	Yes	Yes	Yes	No	Yes	No	Yes	No
Moffatt et al. (2010) [40]	Yes	Yes	Yes	Yes	Yes	No	Yes	No	Yes	Yes
Moffatt et al. (2012) [41]	Yes	Yes	Yes	Yes	Yes	No	Yes	No	Yes	Yes
Moffatt and Noble (2015) [42]	Yes	Yes	Yes	Yes	Yes	No	Yes	No	Yes	Yes
Schröder et al. (2020) [43]	Yes	Yes	Yes	Yes	Yes	No	Yes	Yes	Yes	Yes

Slavova-Azmanova et al. (2020) [44]	Yes	Yes	Yes	NA	Yes	NA	Yes	Yes	Yes	Yes
Timmons et al. (2013a) [45]	Yes	Yes	Yes	Yes	Yes	No	Yes	Yes	Yes	Yes
Timmons et al. (2013b) [46]	Yes	Yes	Yes	Yes	Yes	No	Yes	Yes	Yes	Yes

References

1. National Institutes of Health; National Heart, Lung, and Blood Institute. Study quality assessment tools. Available online: https://www.nhlbi.nih.gov/health-topics/study-quality-assessment-tools (accessed on 28 April 2021).
2. Akechi, T.; Okuyama, T.; Uchida, M.; Nakaguchi, T.; Ito, Y.; Yamashita, H.; Toyama, T.; Komatsu, H.; Kizawa, Y.; Wada, M. Perceived needs, psychological distress and quality of life of elderly cancer patients. *Jpn. J. Clin. Oncol.* 2012, *42*, 704–710, doi:10.1093/jjco/hys075.
3. Arndt, V.; Merx, H.; Stegmaier, C.; Ziegler, H.; Brenner, H. Quality of life in patients with colorectal cancer 1 year after diagnosis compared with the general population: a population-based study. *J. Clin. Oncol.* 2004, *22*, 4829–4836, doi:10.1200/JCO.2004.02.018.
4. Arndt, V.; Koch-Gallenkamp, L.; Jansen, L.; Bertram, H.; Eberle, A.; Holleczek, B.; Schmid-Höpfner, S.; Waldmann, A.; Zeissig, S.R.; Brenner, H. Quality of life in long-term and very long-term cancer survivors versus population controls in Germany. *Acta Oncol.* 2017, *56*, 190–197, doi:10.1080/0284186X.2016.1266089.
5. Arndt, V.; Koch-Gallenkamp, L.; Bertram, H.; Eberle, A.; Holleczek, B.; Pritzkuleit, R.; Waldeyer-Sauerland, M.; Waldmann, A.; Zeissig, S.R.; Doege, D.; et al. Return to work after cancer. A multi-regional population-based study from Germany. *Acta Oncol.* 2019, *58*, 811–818, doi:10.1080/0284186X.2018.1557341.
6. Barbaret, C.; Brosse, C.; Rhondali, W.; Ruer, M.; Monsarrat, L.; Michaud, P.; Schott, A.M.; Delgado-Guay, M.; Bruera, E.; Sanchez, S.; et al. Financial distress in patients with advanced cancer. *PLoS One* 2017, *12*, e0176470, doi:10.1371/journal.pone.0176470.
7. Barbaret, C.; Delgado-Guay, M.O.; Sanchez, S.; Brosse, C.; Ruer, M.; Rhondali, W.; Monsarrat, L.; Michaud, P.; Schott, A.M.; Bruera, E.; et al. Inequalities in Financial Distress, Symptoms, and Quality of Life Among Patients with Advanced Cancer in France and the U.S. *Oncologist* 2019, *24*, 1121–1127, doi:10.1634/theoncologist.2018-0353.
8. Büttner, M.; König, H.-H.; Löbner, M.; Briest, S.; Konnopka, A.; Dietz, A.; Riedel-Heller, S.; Singer, S. Out-of-pocket-payments and the financial burden of 502 cancer patients of working age in Germany: results from a longitudinal study. *Support. Care Cancer* 2019, *27*, 2221–2228, doi:10.1007/s00520-018-4498-1.
9. Doege, D.; Thong, M.S.-Y.; Koch-Gallenkamp, L.; Bertram, H.; Eberle, A.; Holleczek, B.; Pritzkuleit, R.; Waldeyer-Sauerland, M.; Waldmann, A.; Zeissig, S.R.; et al. Health-related quality of life in long-term disease-free breast cancer survivors versus female population controls in Germany. *Breast Cancer Res. Treat.* 2019, *175*, 499–510, doi:10.1007/s10549-019-05188-x.
10. Egestad, H.; Nieder, C. Undesirable financial effects of head and neck cancer radiotherapy during the initial treatment period. *Int. J. Circumpolar Health* 2015, *74*, 26686, doi:10.3402/ijch.v74.26686.
11. Ezeife, D.A.; Morganstein, B.J.; Lau, S.; Law, J.H.; Le, L.W.; Bredle, J.; Cella, D.; Doherty, M.K.; Bradbury, P.; Liu, G.; et al. Financial Burden Among Patients With Lung Cancer in a Publically Funded Health Care System. *Clin. Lung Cancer* 2019, *20*, 231–236, doi:10.1016/j.cllc.2018.12.010.
12. Gordon, L.G.; Walker, S.M.; Mervin, M.C.; Lowe, A.; Smith, D.P.; Gardiner, R.A.; Chambers, S.K. Financial toxicity: a potential side effect of prostate cancer treatment among Australian men. *Eur. J. Cancer Care (Engl)* 2017, *26*, doi:10.1111/ecc.12392.
13. Gordon, L.G.; Beesley, V.L.; Mihala, G.; Koczwara, B.; Lynch, B.M. Reduced employment and financial hardship among middle-aged individuals with colorectal cancer. *Eur. J. Cancer Care (Engl)* 2017, *26*, doi:10.1111/ecc.12744.
14. Gordon, L.G.; Elliott, T.M.; Wakelin, K.; Leyden, S.; Leyden, J.; Michael, M.; Pavlakis, N.; Mumford, J.; Segelov, E.; Wyld, D.K. The Economic Impact on Australian Patients with Neuroendocrine Tumours. *Patient* 2020, *13*, 363–373, doi:10.1007/s40271-020-00412-z.
15. Hanly, P.; Maguire, R.; Ceilleachair, A.O.; Sharp, L. Financial hardship associated with colorectal cancer survivorship: The role of asset depletion and debt accumulation. *Psychooncology.* 2018, *27*, 2165–2171, doi:10.1002/pon.4786.
16. Honda, K.; Gyawali, B.; Ando, M.; Kumanishi, R.; Kato, K.; Sugiyama, K.; Mitani, S.; Masuishi, T.; Narita, Y.; Bando, H.; et al. Prospective Survey of Financial Toxicity Measured by the Comprehensive Score for Financial Toxicity in Japanese Patients With Cancer. *J. Glob. Oncol.* 2019, *5*, 1–8, doi:10.1200/JGO.19.00003.
17. Ivanauskienė, R.; Padaiga, Ž.; Šimoliūnienė, R.; Smailytė, G.; Domeikienė, A. Well-being of newly diagnosed women with breast cancer: which factors matter more? *Support. Care Cancer* 2014, *22*, 519–526, doi:10.1007/s00520-013-2005-2.
18. Kaptein, A.A.; Yamaoka, K.; Snoei, L.; van der Kloot, W.A.; Inoue, K.; Tabei, T.; Kroep, J.R.; Krol-Warmerdam, E.; Ranke, G.; Meirink, C.; et al. Illness perceptions and quality of life in Japanese and Dutch women with breast cancer. *J. Psychosoc. Oncol.* 2013, *31*, 83–102, doi:10.1080/07347332.2012.741092.
19. Koch, L.; Jansen, L.; Herrmann, A.; Stegmaier, C.; Holleczek, B.; Singer, S.; Brenner, H.; Arndt, V. Quality of life in long-term breast cancer survivors - a 10-year longitudinal population-based study. *Acta Oncol.* 2013, *52*, 1119–1128, doi:10.3109/0284186X.2013.774461.
20. Koskinen, J.-P.; Färkkilä, N.; Sintonen, H.; Saarto, T.; Taari, K.; Roine, R.P. The association of financial difficulties and out-of-pocket payments with health-related quality of life among breast, prostate and colorectal cancer patients. *Acta Oncol.* 2019, *58*, 1062–1068, doi:10.1080/0284186X.2019.1592218.

21. Lu, L.; O'Sullivan, E.; Sharp, L. Cancer-related financial hardship among head and neck cancer survivors: Risk factors and associations with health-related quality of life. *Psychooncology.* 2019, *28*, 863–871, doi:10.1002/pon.5034.
22. Mandaliya, H.; Ansari, Z.; Evans, T.; Oldmeadow, C.; George, M. Psychosocial Analysis of Cancer Survivors in Rural Australia: Focus on Demographics, Quality of Life and Financial Domains. *Asian Pac. J. Cancer Prev.* 2016, *17*, 2459–2464.
23. McLean, L.; Hong, W.; McLachlan, S.-A. Financial toxicity in patients with cancer attending a public Australian tertiary hospital: A pilot study. *Asia Pac. J. Clin. Oncol.* 2020, doi:10.1111/ajco.13448.
24. McNeil, R.J.; McCarthy, M.; Dunt, D.; Thompson, K.; Kosola, S.; Orme, L.; Drew, S.; Sawyer, S. Financial Challenges of Cancer for Adolescents and Young Adults and Their Parent Caregivers. *Social Work Research* 2019, *43*, 17–30, doi:10.1093/swr/svy027.
25. Mehlis, K.; Witte, J.; Surmann, B.; Kudlich, M.; Apostolidis, L.; Walther, J.; Jäger, D.; Greiner, W.; Winkler, E.C. The patient-level effect of the cost of Cancer care - financial burden in German Cancer patients. *BMC Cancer* 2020, *20*, 529, doi:10.1186/s12885-020-07028-4.
26. Mercadante, S.; Aielli, F.; Adile, C.; Bonanno, G.; Casuccio, A. Financial distress and its impact on symptom expression in advanced cancer patients. *Support. Care Cancer* 2021, *29*, 485–490, doi:10.1007/s00520-020-05507-9.
27. Paul, C.L.; Hall, A.E.; Carey, M.L.; Cameron, E.C.; Clinton-McHarg, T. Access to care and impacts of cancer on daily life: do they differ for metropolitan versus regional hematological cancer survivors? *J. Rural Health* 2013, *29 Suppl 1*, s43-50, doi:10.1111/jrh.12020.
28. Pearce, A.; Tomalin, B.; Kaambwa, B.; Horevoorts, N.; Duijts, S.; Mols, F.; van de Poll-Franse, L.; Koczwara, B. Financial toxicity is more than costs of care: the relationship between employment and financial toxicity in long-term cancer survivors. *J. Cancer Surviv.* 2019, *13*, 10–20, doi:10.1007/s11764-018-0723-7.
29. Schmidt, M.E.; Scherer, S.; Wiskemann, J.; Steindorf, K. Return to work after breast cancer: The role of treatment-related side effects and potential impact on quality of life. *Eur. J. Cancer Care (Engl)* 2019, *28*, e13051, doi:10.1111/ecc.13051.
30. Sharp, L.; Timmons, A. Pre-diagnosis employment status and financial circumstances predict cancer-related financial stress and strain among breast and prostate cancer survivors. *Support. Care Cancer* 2016, *24*, 699–709, doi:10.1007/s00520-015-2832-4.
31. Zucca, A.; Boyes, A.; Newling, G.; Hall, A.; Girgis, A. Travelling all over the countryside: travel-related burden and financial difficulties reported by cancer patients in New South Wales and Victoria. *Aust. J. Rural Health* 2011, *19*, 298–305, doi:10.1111/j.1440-1584.2011.01232.x.
32. Critical Appraisal Skills Programme. CASP-Qualitative-Checklist 2018.
33. Amir, Z.; Wilson, K.; Hennings, J.; Young, A. The meaning of cancer: implications for family finances and consequent impact on lifestyle, activities, roles and relationships. *Psychooncology.* 2012, *21*, 1167–1174, doi:10.1002/pon.2021.
34. Bennett, J.A.; Brown, P.; Cameron, L.; Whitehead, L.C.; Porter, D.; McPherson, K.M. Changes in employment and household income during the 24 months following a cancer diagnosis. *Support. Care Cancer* 2009, *17*, 1057–1064, doi:10.1007/s00520-008-0540-z.
35. Fitch, M.; Longo, C.J. Exploring the impact of out-of-pocket costs on the quality of life of Canadian cancer patients. *J. Psychosoc. Oncol.* 2018, *36*, 582–596, doi:10.1080/07347332.2018.1486937.
36. Fitch, M.I.; Longo, C.J.; Chan, R.J. Cancer patients' perspectives on financial burden in a universal healthcare system: Analysis of qualitative data from participants from 20 provincial cancer centers in Canada. *Patient Educ. Couns.* 2021, *104*, 903–910, doi:10.1016/j.pec.2020.08.013.
37. Gray, R.E.; James, P.; Manthorne, J.; Gould, J.; Fitch, M.I. A consultation with Canadian rural women with breast cancer. *Health Expect.* 2004, *7*, 40–50, doi:10.1046/j.1369-6513.2003.00248.x.
38. Grinyer, A. The biographical impact of teenage and adolescent cancer. *Chronic Illn.* 2007, *3*, 265–277, doi:10.1177/1742395307085335.
39. Kane, D.; Rajacich, D.; Andary, C. Experiences of cancer patients' return to work. *Can. Oncol. Nurs. J.* 2020, *30*, 113–118, doi:10.5737/23688076302113118.
40. McGrath, P.; Patterson, C.; Yates, P.; Treloar, S.; Oldenburg, B.; Loos, C. A study of postdiagnosis breast cancer concerns for women living in rural and remote Queensland. Part II: Support issues. *Aust. J. Rural Health* 1999, *7*, 43–52, doi:10.1046/j.1440-1584.1999.00216.x.
41. McGrath, P. "It's horrendous--but really, what can you do?" Preliminary findings on financial impact of relocation for specialist treatment. *Aust. Health Rev.* 2000, *23*, 94–103, doi:10.1071/ah000094.
42. Moffatt, S.; Noble, E.; Exley, C. "Done more for me in a fortnight than anybody done in all me life." How welfare rights advice can help people with cancer. *BMC Health Serv. Res.* 2010, *10*, 259, doi:10.1186/1472-6963-10-259.
43. Moffatt, S.; Noble, E.; White, M. Addressing the financial consequences of cancer: qualitative evaluation of a welfare rights advice service. *PLoS One* 2012, *7*, e42979, doi:10.1371/journal.pone.0042979.
44. Moffatt, S.; Noble, E. Work or welfare after cancer? Explorations of identity and stigma. *Sociol. Health Illn.* 2015, *37*, 1191–1205, doi:10.1111/1467-9566.12303.
45. Schröder, S.L.; Schumann, N.; Fink, A.; Richter, M. Coping mechanisms for financial toxicity: a qualitative study of cancer patients' experiences in Germany. *Support. Care Cancer* 2020, *28*, 1131–1139, doi:10.1007/s00520-019-04915-w.
46. Slavova-Azmanova, N.S.; Newton, J.C.; Saunders, C.; Johnson, C.E. 'Biggest factors in having cancer were costs and no entitlement to compensation'-The determinants of out-of-pocket costs for cancer care through the lenses of rural and outer metropolitan Western Australians. *Aust. J. Rural Health* 2020, *28*, 588–602, doi:10.1111/ajr.12686.
47. Timmons, A.; Gooberman-Hill, R.; Sharp, L. "It's at a time in your life when you are most vulnerable": a qualitative exploration of the financial impact of a cancer diagnosis and implications for financial protection in health. *PLoS One* 2013, *8*, e77549, doi:10.1371/journal.pone.0077549.
48. Timmons, A.; Gooberman-Hill, R.; Sharp, L. The multidimensional nature of the financial and economic burden of a cancer diagnosis on patients and their families: qualitative findings from a country with a mixed public-private healthcare system. *Support. Care Cancer* 2013, *21*, 107–117, doi:10.1007/s00520-012-1498-4.

Supplementary material 5: Summary of identified risk factors

Table S5: Summary of identified risk factors

Study	Instrument, validation, domains considered	Definition of dependent variable	Method	Category of risk factors Subcategory	Risk factor as stated in the article	Significance	Effect	Occurrence
Quantitative Studies								
Akechi et al. (2012) [1]	EORTC QLQ-C30<br>Validated<br>Material	Physical condition or medical treatment caused financial difficulties in the past week	ANCOVA	Socio-demographic factors<br>Age	Age >70 years (ref: <70 years)	Significant	-	Before
Arndt et al. (2004) [4]	EORTC-QLQ-C30<br>Validated<br>Material	Physical condition or medical treatment caused financial difficulties in the past week	Not reported	Socio-demographic factors<br>Age	Age <60	Not reported	+	Before
Arndt et al. (2017) [3]	EORTC QLQ-C30<br>Validated<br>Material	Physical condition or medical treatment caused financial difficulties in the past week	ANOVA	Medical (& treatment) factors<br>Time since onset of disease	10+ years post-diagnosis (ref: 5–9 years post-diagnosis,)	Significant	+	After
				Medical (& treatment) factors<br>Time since onset of disease	10+ years post-diagnosis (ref: 5–9 years post-diagnosis,)	Not Significant	-	After
				Medical (& treatment) factors<br>Time since onset of disease	10+ years post-diagnosis (ref: 5–9 years post-diagnosis,)	Significant	+	After
				Medical (& treatment) factors<br>Time since onset of disease	10+ years post-diagnosis (ref: 5–9 years post-diagnosis,)	Significant	+	After
				Medical (& treatment) factors<br>Time since onset of disease	10+ years post-diagnosis (ref: 5–9 years post-diagnosis,)	Not Significant	-	After

Arndt et al. (2019) [2]	EORTC QLQ-C30<br>Validated<br>Material	Physical condition or medical treatment caused financial difficulties in the past week	t-Test	Employment<br>Return to work	Reduction of working hours within 1 year after return to work (ref: no reduction)	Significant	+	After
				Employment<br>Return to work	Quitting Job due to cancer within 5 years after return to work (ref: no quitting)	Significant	+	After
			ANOVA	Employment<br>Return to work	Financial difficulties according to initial return to work status: Return to former work (p-value: comparison of three groups)	Significant	-	After
				Employment<br>Return to work	Financial difficulties according to initial return to work status: Uptake of a new job (p-value: comparison of three groups)	Significant	+	After
				Employment<br>Return to work	Financial difficulties according to initial return to work status: No resumption of work (p-value: comparison of three groups)	Significant	+	After
Barbaret et al. (2017) [5]	Self-developed<br>Not Validated<br>Psychosocial	The cost of treating cancer has caused patient or his/her family distress	Chi²/t-Test (otherwise Mann-Whitney-U-test or Fisher's exact test)	Socio-demographic factors<br>Gender	Gender: Female (ref: Male)	Significant	+	Before
				Socio-demographic factors<br>Age	Age	Significant	-	Before
				Medical (& treatment) factors<br>Time since onset of disease	Average time between diagnosis and study	Not Significant	+	After
				Socio-demographic factors<br>Familial status	Familial Status: Single (ref: Married)	Significant	+	Before
				Socio-demographic factors<br>Education	Education level	Not Significant	~	Before
				Financial resources<br>Pre-diagnosis financial status	Income <15,000 euros yearly (ref: >15,000)	Significant	+	Before

				Medical (& treatment) factors<br>Well-being	ECOG	Not Significant	+	During
				Medical (& treatment) factors<br>Cancer status	Cancer Type Breast Cancer (ref: lung, prostate, colorectal)	Significant	+	During
				Medical (& treatment) factors<br>Cancer status	Cancer stage (metastic, relapsed, locally advanced, other)	Not Significant	~	During
				Medical (& treatment) factors<br>Treatment	Treatment: surgery	Not Significant	±	During
				Medical (& treatment) factors<br>Treatment	Treatment: Chemotherapy	Not Significant	±	During
				Medical (& treatment) factors<br>Treatment	Treatment: Radiotherapy	Not Significant	±	During
				Medical (& treatment) factors<br>Treatment	Treatment: Target therapy	Not Significant	+	During
			Pearson correlation	Financial resources<br>Cancer related financial impact	Subjective financial burden (impact of FD on patients' wellbeing)	Significant	+	During
				Financial resources<br>Cancer related financial impact	Financial concerns	Significant	+	During
				Financial resources<br>Cancer related financial impact	Financial worries	Significant	+	During
				Financial resources<br>Cancer related financial impact	Actual financial difficulties	Significant	+	During
Barbaret et al. (2019) [6]	Self-developed<br>Not Validated<br>Psychosocial	The cost of treating cancer has caused patient or his/her family distress.	Linear regression	Socio-demographic factors<br>Country	Residence of France	Significant	-	Before

				Medical (& treatment) factors<br>Well-being	High HADS-D score	Significant	+	During
				Medical (& treatment) factors<br>Well-being	Lower HADS-D score	Significant	-	During
				Socio-demographic factors<br>Age	Greater Age	Significant	-	Before
				Socio-demographic factors<br>Familial status	Single status	Significant	+	Before
				Medical (& treatment) factors<br>Well-being	Functional and social well-being	Significant	-	During
				Medical (& treatment) factors<br>Cancer status	Metastasis	Significant	+	During
				Medical (& treatment) factors<br>Treatment	Surgery	Significant	+	During
				Medical (& treatment) factors<br>Well-being	Physical well-being	Not Significant	-	During
				Insurance<br>Health insurance	Government insurance	Not Significant	-	During
Büttner et al. (2019) [7]	EORTC QLQ-C30<br>Validated<br>Material	Physical condition or medical treatment caused financial difficulties in the past week	Mean frequencies	Financial resources<br>Cancer related financial impact	Financial burden (end of hospital stay)	Not reported	~	During
				Financial resources<br>Cancer related financial impact	Financial burden (3 month after hospital stay)	Not reported	~	During
				Financial resources<br>Cancer related financial impact	Financial burden (15 month after hospital stay)	Not reported	~	During

				Medical (& treatment) factors<br>Time since onset of disease	Time since diagnosis (end of hospital stay)	Not reported	~	During
				Medical (& treatment) factors<br>Time since onset of disease	Time since diagnosis (3 month after hospital stay)	Not reported	~	During
				Medical (& treatment) factors<br>Time since onset of disease	Time since diagnosis (15 month after hospital stay)	Not reported	~	During
				Financial resources<br>Pre-diagnosis financial status	Income groups < €1000 (ref: >€1000)	Not reported	+	Before
				Financial resources<br>Pre-diagnosis financial status	Income groups < €1000 (ref: >€1000)	Not reported	+	Before
				Financial resources<br>Pre-diagnosis financial status	Income groups < €1000 (ref: >€1000)	Not reported	+	Before
Doege et al. (2019) [8]	EORTC QLQ-C30<br>Validated<br>Material	Physical condition or medical treatment caused financial difficulties in the past week	Linear regression	Medical (& treatment) factors<br>Time since onset of disease	≥ 10 years post-diagnosis (ref: 5–9 years post-diagnosis)	Not Significant	+	After
				Medical (& treatment) factors<br>Time since onset of disease	5–9 years post-diagnosis (ref: control)	Significant	+	After
				Medical (& treatment) factors<br>Time since onset of disease	≥ 10 years post-diagnosis (ref: control)	Significant	+	After
				Medical (& treatment) factors<br>Time since onset of disease	≥ 10 years post-diagnosis (ref: 5–9 years post-diagnosis)	Not Significant	-	After
				Medical (& treatment) factors<br>Time since onset of disease	5–9 years post-diagnosis (ref: control)	Significant	+	After
				Medical (& treatment) factors<br>Time since onset of disease	≥ 10 years post-diagnosis (ref: control)	Not Significant	+	After

				Medical (& treatment) factors<br>Time since onset of disease	≥ 10 years post-diagnosis (ref: 5–9 years post-diagnosis)	Not Significant	+	After
				Medical (& treatment) factors<br>Time since onset of disease	5–9 years post-diagnosis (ref: control)	Not Significant	+	After
				Medical (& treatment) factors<br>Time since onset of disease	≥ 10 years post-diagnosis (ref: control)	Not Significant	+	After
				Medical (& treatment) factors<br>Time since onset of disease	≥ 10 years post-diagnosis (ref: 5–9 years post-diagnosis)	Not Significant	+	After
				Medical (& treatment) factors<br>Time since onset of disease	5–9 years post-diagnosis (ref: control)	Not Significant	+	After
				Medical (& treatment) factors<br>Time since onset of disease	≥ 10 years post-diagnosis (ref: control)	Not Significant	+	After
				Medical (& treatment) factors<br>Time since onset of disease	≥ 10 years post-diagnosis (ref: 5–9 years post-diagnosis)	Not Significant	-	After
				Medical (& treatment) factors<br>Time since onset of disease	5–9 years post-diagnosis (ref: control)	Not Significant	+	After
				Medical (& treatment) factors<br>Time since onset of disease	≥ 10 years post-diagnosis (ref: control)	Not Significant	-	After
Egestad et al. (2015) [9]	EORTC QLQ-C30, EORTC QLQ-H&N35<br>Validated<br>Material	Physical condition or medical treatment caused financial difficulties in the past week	t-Test	Socio-demographic factors<br>Gender	Men (ref: women)	Not Significant	-	Before
				Socio-demographic factors<br>Gender	Men (ref: women)	Not Significant	+	Before

Socio-demographic factors<br>Gender	Men (ref: women)	Not Significant	-	Before
Medical (& treatment) factors<br>Time since onset of disease	Time since treatment: baseline (ref: end)	Not Significant	-	During
Medical (& treatment) factors<br>Time since onset of disease	Time since treatment: baseline (ref: end)	Not Significant	+	During
Socio-demographic factors<br>Age	Age <65 years (ref: >65 years)	Not Significant	-	Before
Socio-demographic factors<br>Age	Age <65 years (ref: >65 years)	Not Significant	+	Before
Socio-demographic factors<br>Age	Age <65 years (ref: >65 years)	Not Significant	±	Before
Medical (& treatment) factors<br>Time since onset of disease	Time since treatment baseline (ref: end)	Not Significant	-	During
Medical (& treatment) factors<br>Time since onset of disease	Time since treatment baseline (ref: end)	Not Significant	+	During
Socio-demographic factors<br>Familial status	Marital status married (ref: single)	Not Significant	±	Before
Socio-demographic factors<br>Familial status	Marital status married (ref: single)	Not Significant	-	Before
Socio-demographic factors<br>Familial status	Marital status married (ref: single)	Not Significant	+	Before
Medical (& treatment) factors<br>Time since onset of disease	Time since treatment baseline (ref: end)	Not Significant	+	During

				Medical (& treatment) factors<br>Time since onset of disease	Time since treatment baseline (ref: end)	Not Significant	-	During
				Medical (& treatment) factors<br>Treatment	Treatment RT alone (ref: Cisplatin and RT)	Not Significant	-	During
				Medical (& treatment) factors<br>Treatment	Treatment RT alone (ref: Cisplatin and RT)	Not Significant	+	During
				Medical (& treatment) factors<br>Treatment	Treatment RT alone (ref: Cisplatin and RT)	Not Significant	+	During
				Medical (& treatment) factors<br>Time since onset of disease	Time since treatment baseline (ref: end)	Not Significant	-	During
				Medical (& treatment) factors<br>Time since onset of disease	Time since treatment baseline (ref: end)	Not Significant	+	During
Ezeife et al. (2019) [10]	COST<br>Validated<br>Material, Psychosocial	Financial toxicity measured by the score of the COST.<br>Dichotomisation of the score for logistic regression (cut off: <21)	Logistic regression	Socio-demographic factors<br>Age	Age <65 (ref: >65)	Significant	+	Before
				Financial spending<br>Cancer-related total costs	Total OOP 1000-5000$ (ref: <1000$)	Significant	+	During
				Financial spending<br>Cancer-related total costs	Total OOP 5000-10000$ (ref: <1000$)	Significant	+	During
				Financial spending<br>Cancer-related total costs	Total OOP >10000$ (ref: <1000$)	Significant	+	During
				Insurance<br>Health insurance	Private health insurance (ref:No)	Significant	-	Before

				Socio-demographic factors<br>Geography	Distance from Cancer centre >17 (ref: <17)	Not Significant	-	During
				Medical (& treatment) factors<br>Treatment	Systemic therapy - not on clinical trial (ref: Clinical trial)	Not Significant	+	During
				Financial resources<br>Pre-diagnosis financial status	Annual income <40.000$ (ref: >80.000$)	Not Significant	+	Before
				Financial resources<br>Pre-diagnosis financial status	Annual income 40-80.000$ (ref: >80.000$)	Not Significant	+	Before
Gordon et al. (2017a) [11]	Self-developed<br>Not Validated<br>Material, Psychosocial, Behavioural	See questions asked	Frequencies	Financial spending<br>Consequences of OOP	Decided against treatment due to high expenses			During
				Financial spending<br>Consequences of OOP	Spend more for their cancer treatment than they expected			During
				Medical (& treatment) factors<br>Cancer status	Prostate cancer had caused them ‘a great deal’ of distress			During
				Financial resources<br>Cancer related financial impact	"Just getting" on or "struggeling financially"			During
				Financial resources<br>Coping	Drawn on savings to pay for treatment			During
				Financial resources<br>Coping	Sold assets to pay for treatment			During
				Financial resources<br>Coping	Increased a credit card limit to pay for treatment			During

			Not reported	Medical (& treatment) factors<br>Time since onset of disease	Recently diagnosed with cancer (ref: responses of all respondents)	Not reported	+	During
Gordon et al. (2017b) [12]	Self-developed<br>Not Validated<br>Material, Behavioural	Financial hardship: Perceived prosperity, Financial strain, Ability to raise money	Logistic regression	Financial resources<br>Cancer related financial impact	Not financially comfortable	Significant	+	During
				Financial resources<br>Cancer related financial impact	Action indicated financial strain	Not Significant	+	During
				Financial resources<br>Cancer related financial impact	Unable to raise money easily	Not Significant	+	During
Gordon et al. (2020) [13]	Self-developed, COST<br>Material, Psychosocial, Behavioural	Financial toxicity measured by the score of the COST<br>Questions about emplyoment, income in relation to distress, treatment decisions and personal distress	Frequencies	Financial spending<br>Consequences of OOP	Spending more money on the cost of treating cancer than expected			During
				Social circumstances<br>Family	Family distress			During
				Financial spending<br>Consequences of OOP	Cost was a consideration in choosing their cancer treatment course			During
				Financial spending<br>Consequences of OOP	Not proceed with treatment due to cost			During
Hanly et al. (2018) [14]	Self-developed<br>Not Validated<br>Psychosocial	How the patient felt about his/her household's financial situation	Logistic regression	Medical (& treatment) factors<br>Treatment	Stoma (ref: no stoma)	Significant	+	During
				Socio-demographic factors<br>Familial status	Dependents (ref: no dependants)	Significant	+	Before

				Social circumstances<br>Support	Obtaining help from family members and friends (ref: no help)	Significant	+	During
				Financial resources<br>Coping	Using saving (ref: savings, but not used)	Significant	+	During
				Financial resources<br>Coping	No savings (ref: savings not used)	Significant	+	During
Honda et al. (2019) [15]	COST<br>Validated<br>Material, Psychosocial	Financial toxicity measured by the score of the COST	Linear regression	Socio-demographic factors<br>Age	Age (in years)	Significant	-	Before
				Medical (& treatment) factors<br>Cancer status	Local cancer stage (ref: metastatic)	Not Significant	-	During
				Medical (& treatment) factors<br>Time since onset of disease	Time to first chemotherapy, month	Not Significant	+	During
				Insurance<br>Health insurance	Private health insurance (ref: no)	Not Significant	-	Before
				Medical (& treatment) factors<br>Cancer status	Number of admissions last year	Not Significant	+	During
				Financial spending<br>Consequences of OOP	Use of strategies to cope with cost of cancer care expenses: Yes (ref: No)	Significant	+	During
				Socio-demographic factors<br>Familial status	Marital status: Nonmarried (ref: Married)	Not Significant	+	Before
				Employment<br>Pre-diagnosis work status	Employment status: Part time (ref: Full time)	Significant	+	During
				Employment<br>Pre-diagnosis work status	Employment status: Retired, not because of cancer (ref: Full time)	Not Significant	+	During

	Employment<br>Pre-diagnosis work status	Employment status: Retired because of cancer (ref: Full time)	Significant	+	During
	Employment<br>Pre-diagnosis work status	Employment status: Leave of absence (ref: Full time)	Not Significant	+	During
	Employment<br>Pre-diagnosis work status	Employment status: Not working outside home (ref: Full time)	Not Significant	+	During
	Financial resources<br>Pre-diagnosis financial status	Household income per year ¥2,000,000-3,999,999 (ref: <¥2,000,000)	Not Significant	+	Before
	Financial resources<br>Pre-diagnosis financial status	Household income per year ¥ 4,000,000-5,999,999 (ref: <¥2,000,000)	Not Significant	+	Before
	Financial resources<br>Pre-diagnosis financial status	Household income per year ¥ 6,000,000-7,999,999 (ref: <¥2,000,000)	Not Significant	+	Before
	Financial resources<br>Pre-diagnosis financial status	Household income per year ¥ 8,000,000-9,999,999 (ref: <¥2,000,000)	Not Significant	-	Before
	Financial resources<br>Pre-diagnosis financial status	Household income per year ¥ >10,000,000 (ref: <¥2,000,000)	Not Significant	+	Before
	Financial resources<br>Pre-diagnosis financial status	Household savings ¥ 2,000,000-3,999,999 (ref: <¥2,000,000)	Not significant	+	Before
	Financial resources<br>Pre-diagnosis financial status	Household savings ¥ 4,000,000-5,999,999 (ref: <¥2,000,000)	Significant	-	Before
	Financial resources<br>Pre-diagnosis financial status	Household savings ¥ 6,000,000-7,999,999 (ref: <¥2,000,000)	Not Significant	+	Before
	Financial resources<br>Pre-diagnosis financial status	Household savings ¥ 8,000,000-9,999,999 (ref: <¥2,000,000)	Significant	-	Before

				Financial resources<br>Pre-diagnosis financial status	Household savings ¥ 10,000,000-14,999,999 (ref: <¥2,000,000)	Significant	-	Before
				Financial resources<br>Pre-diagnosis financial status	Household savings ¥ >15,000,000 (ref: <¥2,000,000)	Significant	-	Before
Ivanauskienė et al. (2014) [16]	EORTC QLQ-C30<br>Validated<br>Material	Physical condition or medical treatment caused financial difficulties in the past week	Logistic regression	Medical (& treatment) factors<br>Treatment	Underwend psychological counseling (ref: Did not seek for psychological counseling)	Significant	-	During
				Employment<br>Pre-diagnosis work status	Not working (unemployed, retired, or being disabled by breast cancer) (ref: Working)	Significant	+	During
				Medical (& treatment) factors<br>Well-being	Comorbidities >2 (ref: Comorbidities <2)	Significant	+	During
				Medical (& treatment) factors<br>Treatment	Surgery, radiotherapy, chemotherapy, and hormone therapy (ref: No)	Significant	+	During
Kaptein et al. (2013) [17]	EORTC QLQ-C30<br>Validated<br>Material	Physical condition or medical treatment caused financial difficulties in the past week	t-test or ANOVA	Socio-demographic factors<br>Country	Country: Japan (ref: Netherlands)	Significant	+	Before
				Medical (& treatment) factors<br>Time since onset of disease	Occasion (Japan)	Not Significant	-	During
				Medical (& treatment) factors<br>Time since onset of disease	Occasion (Netherlands)	Not Significant	+	During
Koch et al. (2013) [18]	EORTC QLQ-C30<br>Validated<br>Material	Physical condition or medical treatment caused financial difficulties in the past week	ANOVA	Socio-demographic factors<br>Age	Age <49	Significant	+	Before
				Socio-demographic factors<br>Age	Age 50 - 64	Significant	+	Before

Socio-demographic factors<br>Age	Age >65	Significant	+	Before
Medical (& treatment) factors<br>Time since onset of disease	Time since diagnosis - 3 years since diagnosis (ref: 1 year since diagnosis)	Significant	-	During
Medical (& treatment) factors<br>Time since onset of disease	Time since diagnosis - 5-7 years since diagnosis (ref: 3 year since diagnosis)	Significant	+	During
Medical (& treatment) factors<br>Time since onset of disease	Time since diagnosis - 10 years since diagnosis (ref: 3 year since diagnosis)	Significant	+	After
Medical (& treatment) factors<br>Time since onset of disease	Comparison over time for time since diagnosis	Significant	~	After
Medical (& treatment) factors<br>Time since onset of disease	Time since diagnosis - 3 years since diagnosis (ref: 1 year since diagnosis)	Significant	-	During
Medical (& treatment) factors<br>Time since onset of disease	Time since diagnosis - 5-7 years since diagnosis (ref: 3 year since diagnosis)	Significant	+	After
Medical (& treatment) factors<br>Time since onset of disease	Time since diagnosis - 10 years since diagnosis (ref: 3 year since diagnosis)	Significant	+	After
Medical (& treatment) factors<br>Time since onset of disease	Survivor (ref: control)	Significant	+	During
Medical (& treatment) factors<br>Time since onset of disease	Survivor (ref: control)	Significant	+	During
Medical (& treatment) factors<br>Time since onset of disease	Survivor (ref: control)	Significant	+	After
Medical (& treatment) factors<br>Time since onset of disease	Survivor (ref: control)	Significant	+	After

				Medical (& treatment) factors<br>Time since onset of disease	Comparison over time for time since diagnosis	Significant	~	After
Koskinen et al. (2019) [19]	EORTC-QLQ-C30<br>Validated<br>Material	Physical condition or medical treatment caused financial difficulties in the past week	Linear regression	Socio-demographic factors<br>Age	Age	Significant	-	Before
				Socio-demographic factors<br>Familial status	Cohabiting	Significant	-	Before
				Socio-demographic factors<br>Education	High education	Significant	-	Before
				Financial spending<br>Cancer-related total costs	OOP payments	Significant	+	During
				Financial spending<br>Cancer-related total costs	Total costs	Significant	+	During
				Employment<br>Pre-diagnosis work status	Unemployment	Significant	+	Before
Lu et al. (2019) [20]	Self-designed<br>Not Validated<br>Psychosocial	Feeling about household financial situation as a result of cancer.	Poisson regression	Socio-demographic factors<br>Age	Age: 50-64 (ref: <50)	Not significant	-	Before
				Socio-demographic factors<br>Age	Age: >64 (ref: <50)	Significant	-	Before
				Financial resources<br>Pre-diagnosis financial status	Pre-diagnosis financial stress - difficult (ref: easy)	Significant	+	Before
				Medical (& treatment) factors<br>Treatment	Initial treatment: Surgery + radiotherapy (ref: Surgery only)	Not significant	±	During

				Medical (& treatment) factors<br>Treatment	Initial treatment: Radiotherapy only (ref: surgery only)	Not significant	+	During
				Medical (& treatment) factors<br>Treatment	Initial treatment: Chemotherapy ± radiotherapy (ref: surgery only)	Not significant	+	During
				Medical (& treatment) factors<br>Treatment	Initial treatment: Chemotherapy + surgery ± radiotherapy (ref: surgery only)	Significant	+	During
				Medical (& treatment) factors<br>Treatment	Initial treatment: Other (ref: surgery only)	Not significant	-	During
Mandaliya et al. (2016) [21]	QLACS, HILDA<br>Validated<br>Material	Financial stress score of the HILDA	Linear regression	Socio-demographic factors<br>Gender	Gender	Not significant	-	Before
				Socio-demographic factors<br>Age	Age	Not significant	-	Before
				Socio-demographic factors<br>Country	Birth Country	Not significant	+	Before
				Socio-demographic factors<br>Familial status	Marital status	Not significant	+	Before
				Socio-demographic factors<br>Familial status	Number of children living in your care	Not significant	+	Before
				Financial resources<br>Pre-diagnosis financial status	Income	Not significant	-	Before
				Insurance<br>Health insurance	Health insurance	Significant	-	Before
				Medical (& treatment) factors<br>Treatment	Number of treatments	Not significant	-	During

				Employment<br>Pre-diagnosis work status	Employment: Retired (ref: Employed)	Not significant	-	After
				Employment<br>Pre-diagnosis work status	Employment: Not employed (ref: Employed)	Not significant	+	After
				Socio-demographic factors<br>Education	Education: No formal education & other (ref: High school)	Not significant	-	Before
				Socio-demographic factors<br>Education	Education:University or Vocational (ref: High school)	Not significant	-	Before
				Medical (& treatment) factors<br>Cancer status	Cancer type: Colon/rectum/bowel (ref: breast)	Not significant	-	During
				Medical (& treatment) factors<br>Cancer status	Cancer type: Other (ref: breast)	Not significant	+	During
McLean et al. (2020) [22]	COST, iPCQ<br>Validated<br>Material, Psychosocial	Financial toxicity measured by the score of the COST.	Linear regression	Socio-demographic factors<br>Age	Age	Significant	-	Before
				Socio-demographic factors<br>Gender	Sex: Female (ref: Male)	Not significant	+	Before
				Socio-demographic factors<br>Geography	ARIA-Index	Not significant	-	During
				Financial resources<br>Pre-diagnosis financial status	Income 50 000 to 75 000	Not significant	+	Before
				Financial resources<br>Pre-diagnosis financial status	Income 75 000 to 100 000	Not significant	+	Before
				Financial resources<br>Pre-diagnosis financial status	Income >100 000	Not significant	+	Before

McNeil et al. (2019) [23]	AYA survey<br>Not Validated<br>Material	The experience of any financial difficulties as a consequence of cancer diagnosis and treatment	Logistic regression	Socio-demographic factors<br>Gender	Gender: Male (ref: Female)	Not significant	-	Before
				Socio-demographic factors<br>Geography	Geography: Regional/rural (ref: Metropolitan)	Not significant	+	Before
				Socio-demographic factors<br>Familial status	Living arrangement: Family home (ref: Living outside family home)	Significant	-	Before
				Employment<br>Pre-diagnosis work status	Employment status: Unemployed (ref: Employed)	Not significant	+	During
				Socio-demographic factors<br>Age	Age at diagnosis: 20-25 (ref: 15-19 years)	Significant	+	Before
				Medical (& treatment) factors<br>Treatment	Length of hospital stay: Long stay (ref: short stay)	Not significant	+	During
				Medical (& treatment) factors<br>Treatment	On/off treatment: On treatment (ref: Off treatment)	Not significant	+	During
				Medical (& treatment) factors<br>Treatment	Treatment setting: Adult (ref: Pediatric)	Not significant	-	During
				Medical (& treatment) factors<br>Cancer status	Cancer type: Blood cancer (ref: Other)	Not significant	-	During
Mehlis et al. (2020) [24]	Self-developed, NCCN Distress Thermometer<br>Not Validated<br>Material, Psychosocial, Behavioural	How much one has been distressed in the last week, including today	Linear regression	Medical (& treatment) factors<br>Cancer status	Primary tumor: Neuroendocrine (ref: Colorectal)	Not significant	+	During
				Socio-demographic factors<br>Gender	Sex: Female (ref: Male)	Significant	+	Before

				Socio-demographic factors<br>Age	Age	Not significant	±	Before
				Socio-demographic factors<br>Familial status	Number of children living in household	Not significant	-	Before
				Employment<br>Pre-diagnosis work status	Unemployed	Not significant	-	During
				Employment<br>Pre-diagnosis work status	Retired	Not significant	-	During
				Insurance<br>Health insurance	Private health insurance	Significant	-	Before
				Medical (& treatment) factors<br>Treatment	Intensity of therapy	Not significant	+	During
				Medical (& treatment) factors<br>Treatment	Duration of therapy	Not significant	-	During
				Medical (& treatment) factors<br>Treatment	Duration of illness	Not significant	+	During
				Financial resources<br>Cancer related financial impact	Financial loss relative to income	Significant	+	During
Mercadante et al. (2020) [25]	Financial distress item within the ESAS<br>Not Validated<br>Psychosocial	Having more financial distress than psychological distress or distress about physical functioning or social/family distress or emotional distress	Linear regression	Socio-demographic factors<br>Age	Age	Significant	+	Before
				Socio-demographic factors<br>Familial status	Marital status: Divorced or separated (ref: n.s.)	Significant	+	Before

				Medical (& treatment) factors<br>Well-being	ESAS anxiety	Significant	+	During
				Medical (& treatment) factors<br>Well-being	Total ESAS	Significant	+	During
				Medical (& treatment) factors<br>Well-being	Poor social/family well-being	Significant	+	During
				Medical (& treatment) factors<br>Well-being	HADS-A	Significant	+	During
				Medical (& treatment) factors<br>Well-being	Global HADS	Significant	+	During
Paul et al. (2013) [26]	Self-developed<br>Not Validated<br>Material	Since receiving diagnosis, did any of the following occur as a result of having cancer tests or treatment: (a) "Had to take time off work," (b) "Had less income," (c) "Had to leave work or close business," (d) "Had difficulty with bills or other payments," (e) "Had trouble with day to day expenses," (f) "Used up your savings,"	Chi²-Test	Employment<br>Direct implications	Had to take time off work - Metropolitan region (ref: non-metropolitan)	Not significant	+	During
				Employment<br>Direct implications	Had to leave work or close business - Metropolitan region (ref: non-metropolitan)	Not significant	+	During
				Financial resources<br>Pre-diagnosis financial status	Had less income - Metropolitan region (Ref: non-metropolitan)	Significant	+	During
				Financial resources<br>Cancer related financial impact	Had difficulty with bills or other payments - Metropolitan region (ref: non-metropolitan)	Not significant	+	During
				Financial resources<br>Coping	Used up savings - Metropolitan region (ref: non-metropolitan)	Not significant	+	During

				Financial resources<br>Cancer related financial impact	Had trouble with day-to-day expenses - Metropolitan region (ref: non-metropolitan)	Not significant	+	During
				Socio-demographic factors<br>Geography	Living in a major city (ref: non-metropolitan)	Significant	+	Before
Pearce et al. (2019) [27]	EORTC QLQ-C30<br>Validated<br>Material	Physical condition or medical treatment caused financial difficulties in the past week	Logistic regression	Socio-demographic factors<br>Age	Age in years	Significant	+	Before
				Socio-demographic factors<br>Age	Age²	Significant	-	Before
				Socio-demographic factors<br>Familial status	Marital status: Married (ref: Not married)	Significant	-	Before
				Socio-demographic factors<br>Education	Education: High education (ref: Low education)	Significant	-	Before
				Socio-demographic factors<br>Socioeconomic status	Socioeconomic status: Medium SES (ref: low SES)	Significant	-	Before
				Socio-demographic factors<br>Socioeconomic status	Socioeconomic status: High SES (ref: low SES)	Significant	-	Before
				Employment<br>Pre-diagnosis work status	Employment: Paid employment (ref: No paid employment)	Not Significant	-	After
				Medical (& treatment) factors<br>Cancer status	Tumour type: Colorectal (ref: Basal cell carcinoma)	Significant	+	During
				Medical (& treatment) factors<br>Cancer status	Tumour type: Prostate (ref: Basal cell carcinoma)	Not Significant	+	During
				Medical (& treatment) factors<br>Cancer status	Tumour type: Gynaecological (ref: Basal cell carcinoma)	Not Significant	+	During

		Medical (& treatment) factors<br>Cancer status	Tumour type: Haematological (ref: Basal cell carcinoma)	Not Significant	+	During
		Medical (& treatment) factors<br>Cancer status	Tumour type: Melanoma (ref: Basal cell carcinoma)	Not Significant	-	During
		Medical (& treatment) factors<br>Cancer status	Tumour type: Thyroid (ref: Basal cell carcinoma)	Not Significant	+	During
		Socio-demographic factors<br>Gender	Gender: Male (ref: female)	Significant	+	Before
		Medical (& treatment) factors<br>Well-being	Physical functioning	Not Significant	-	During
		Medical (& treatment) factors<br>Well-being	Role functioning	Not Significant	-	After
		Medical (& treatment) factors<br>Well-being	Emotional functioning	Significant	-	After
		Medical (& treatment) factors<br>Well-being	Cognitive functioning	Significant	-	After
		Medical (& treatment) factors<br>Well-being	Social functioning	Significant	-	After
		Medical (& treatment) factors<br>Well-being	Global health status/QoL	Not Significant	-	After
		Medical (& treatment) factors<br>Side effect	Fatigue	Not Significant	-	After
		Medical (& treatment) factors<br>Side effect	Nausea/vomiting	Not Significant	-	After

Medical (& treatment) factors<br>Side effect	Pain	Not Significant	+	After
Medical (& treatment) factors<br>Side effect	Dyspnoea	Not Significant	+	After
Medical (& treatment) factors<br>Side effect	Insomnia	Not Significant	+	After
Medical (& treatment) factors<br>Side effect	Appetite loss	Not Significant	-	After
Medical (& treatment) factors<br>Side effect	Constipation	Not Significant	+	After
Medical (& treatment) factors<br>Side effect	Diarrhoea	Not Significant	-	After
Medical (& treatment) factors<br>Time since onset of disease	Time since diagnosis	Not Significant	-	During/After
Employment<br>Return to work	Interaction: time since diagnosis x employment	Significant	+	After
Socio-demographic factors<br>Age	Age in years	Significant	+	Before
Socio-demographic factors<br>Age	Age^2	Significant	-	Before
Socio-demographic factors<br>Familial status	Marital status: Married (ref: Not married)	Not Significant	-	Before
Socio-demographic factors<br>Education	Education: High education (ref: Low education)	Not Significant	-	Before

Socio-demographic factors<br>Socioeconomic status	Socioeconomic status: Medium SES (ref: low SES)	Significant	-	Before
Socio-demographic factors<br>Socioeconomic status	Socioeconomic status: High SES (ref: low SES)	Significant	-	Before
Employment<br>Pre-diagnosis work status	Employment: Paid employment (ref: No paid employment)	Significant	-	After
Medical (& treatment) factors<br>Cancer status	Tumour type: Colorectal (ref: Basal cell carcinoma)	Significant	+	During
Medical (& treatment) factors<br>Cancer status	Tumour type: Prostate (ref: Basal cell carcinoma)	Not Significant	+	During
Medical (& treatment) factors<br>Cancer status	Tumour type: Gynaecological (ref: Basal cell carcinoma)	Not Significant	+	During
Medical (& treatment) factors<br>Cancer status	Tumour type: Haematological (ref: Basal cell carcinoma)	Not Significant	+	During
Medical (& treatment) factors<br>Cancer status	Tumour type: Melanoma (ref: Basal cell carcinoma)	Not Significant	+	During
Medical (& treatment) factors<br>Cancer status	Tumour type: Thyroid (ref: Basal cell carcinoma)	Not Significant	+	During
Socio-demographic factors<br>Gender	Gender: Male (ref: female)	Significant	+	Before
Medical (& treatment) factors<br>Well-being	Physical functioning	Not Significant	+	After
Medical (& treatment) factors<br>Well-being	Role functioning	Not Significant	-	After

		Medical (& treatment) factors<br>Well-being	Emotional functioning	Significant	-	After
		Medical (& treatment) factors<br>Well-being	Cognitive functioning	Significant	-	After
		Medical (& treatment) factors<br>Well-being	Social functioning	Significant	-	After
		Medical (& treatment) factors<br>Well-being	Global health status/QoL	Not Significant	-	After
		Medical (& treatment) factors<br>Side effect	Fatigue	Not Significant	+	After
		Medical (& treatment) factors<br>Side effect	Nausea/vomiting	Not Significant	-	After
		Medical (& treatment) factors<br>Side effect	Pain	Not Significant	-	After
		Medical (& treatment) factors<br>Side effect	Dyspnoea	Not Significant	+	After
		Medical (& treatment) factors<br>Side effect	Insomnia	Not Significant	+	After
		Medical (& treatment) factors<br>Side effect	Appetite loss	Not Significant	-	After
		Medical (& treatment) factors<br>Side effect	Constipation	Not Significant	+	After
		Medical (& treatment) factors<br>Side effect	Diarrhoea	Not Significant	-	After

				Medical (& treatment) factors<br>Time since onset of disease	Time since diagnosis	Not Significant	-	During/After
				Employment<br>Return to work	Interaction: time since diagnosis x employment	Not Significant	+	During/After
Schmidt et al. (2019) [28]	EORTC QLQ-C30<br>Validated<br>Material	Physical condition or medical treatment caused financial difficulties in the past week	Frequencies	Employment<br>Return to work	Cessation of work	Significant	+	After
			Mean values	Employment<br>Return to work	Working status discountinued (ref: reduced working hours)	Not reported	±	During
				Employment<br>Return to work	Working status discountinued (ref: reduced working hours)	Not reported	±	During
				Employment<br>Return to work	Working status discountinued (ref: reduced working hours)	Not reported	+	After
				Employment<br>Return to work	Working status reduced working hours (ref: worked as before diagnosis)	Not reported	+	During
				Employment<br>Return to work	Working status reduced working hours (ref: worked as before diagnosis)	Not reported	+	During
				Employment<br>Return to work	Working status reduced working hours (ref: worked as before diagnosis)	Not reported	+	After
			ANCOVA	Employment<br>Return to work	Working status over time discountinued (ref: worked as before diagnosis)	Significant	+	After
Sharp et al. (2016) [29]	Self-developed<br>Not Validated<br>Material	The impact of the cancer diagnosis on the household's ability to make ends meet	Poisson regression	Socio-demographic factors<br>Gender	Female: breast cancer (ref: Male: prostate cancer)	Significant	-	During

Socio-demographic factors<br>Familial status	Dependants: One or more (ref: None)	Significant	+	Before
Employment<br>Pre-diagnosis work status	Employment status at diagnosis: Self-employed (ref: Working for employer)	Not Significant	+	Before
Employment<br>Pre-diagnosis work status	Employment status at diagnosis: Not working (ref: Working for employer)	Significant	-	Before
Employment<br>Pre-diagnosis work status	Employment status at diagnosis: Retired (ref: Working for employer)	Significant	-	Before
Financial resources<br>Pre-diagnosis financial status	Household income pre-diagnosis: Second quartile (ref: Lowest quartile)	Significant	-	Before
Financial resources<br>Pre-diagnosis financial status	Household income pre-diagnosis: Third quartile (ref: Lowest quartile)	Significant	-	Before
Financial resources<br>Pre-diagnosis financial status	Household income pre-diagnosis: Highest quartile (ref: Lowest quartile)	Not Significant	-	Before
Financial resources<br>Pre-diagnosis financial status	Household income pre-diagnosis: Not stated (ref: Lowest quartile)	Not Significant	-	Before
Financial resources<br>Pre-diagnosis financial status	Financial stress pre-diagnosis (household ability to make ends meet): Fairly easy (ref: Easy)	Significant	+	Before
Financial resources<br>Pre-diagnosis financial status	Financial stress pre-diagnosis (household ability to make ends meet): Difficult (ref: Easy)	Significant	+	Before
Financial resources<br>Financial commitment	Had a mortgage and/or personal loan(s): Yes (ref: No)	Significant	+	Before
Insurance<br>Health insurance	Medical card: Held one at diagnosis (ref: No card)	Not Significant	+	Before

			Insurance<br>Health insurance	Medical card: Obtained following diagnosis (ref: No Card)	Significant	+	Before
			Insurance<br>Health insurance	Private health insurance at diagnosis: Yes (ref: No)	Significant	-	Before
			Financial spending<br>Direct medical costs	Direct (cancer related) medical costs: Yes, lowest tertile (≤€400) (ref: None reported)	Not Significant	+	During
			Financial spending<br>Direct medical costs	Direct (cancer related) medical costs: Yes, middle tertile (€401–€1100) (ref: None reported)	Not Significant	+	During
			Financial spending<br>Direct medical costs	Direct (cancer related) medical costs: Yes, highest tertile (>€1100) (ref: None reported)	Significant	+	During
			Financial spending<br>Direct non-medical costs	Increased household bills: Yes (ref: No)	Significant	+	During
Self-developed<br>Not Validated<br>Psychosocial	Feeling about household financial situation as a result of cancer	Poisson regression	Socio-demographic factors<br>Familial status	Dependants: One or more (ref: None)	Significant	+	Before
			Employment<br>Pre-diagnosis work status	Employment status at diagnosis: Self-employed (ref: Working for employer)	Not Significant	-	Before
			Employment<br>Pre-diagnosis work status	Employment status at diagnosis: Not working (ref: Working for employer)	Significant	-	Before
			Employment<br>Pre-diagnosis work status	Employment status at diagnosis: Retired (ref: Working for employer)	Significant	-	Before
			Financial resources<br>Pre-diagnosis financial status	Financial stress pre-diagnosis (household ability to make ends meet): Fairly easy (ref: Easy)	Significant	+	Before

				Financial resources<br>Pre-diagnosis financial status	Financial stress pre-diagnosis (household ability to make ends meet): Difficult (ref: Easy)	Significant	+	Before
				Insurance<br>Health insurance	Medical card: Held one at diagnosis (ref: No card)	Not Significant	-	Before
				Insurance<br>Health insurance	Medical card: Obtained following diagnosis (ref: No Card)	Significant	+	Before
				Financial spending<br>Direct medical costs	Direct (cancer related) medical costs: Yes, lowest tertile (≤€400) (ref: None reported)	Significant	-	During
				Financial spending<br>Direct medical costs	Direct (cancer related) medical costs: Yes, middle tertile (€401–€1100) (ref: None reported)	Not Significant	-	During
				Financial spending<br>Direct medical costs	Direct (cancer related) medical costs: Yes, highest tertile (>€1100) (ref: None reported)	Not Significant	-	During
Zucca et al. (2011) [30]	EORTC QLQ-C30, Supportive Care Needs Survey<br>Validated<br>Material	Physical condition or medical treatment caused financial difficulties in the past week	Logistic regression	Socio-demographic factors<br>Geography	Travel for cancer treatment: More than 2 hours (ref: Less than 2 hours)	Significant	+	During
				Socio-demographic factors<br>Geography	Lived away from home for treatment: Lived away (ref: Did not live away)	Significant	+	During
Qualitative studies								
Amir (2012) [31]	Interviews	The combined effect of lost income and increased expenditure that is problematic to patients and families	Exploratory qualitative analysis of individual Interviews	Knowledge<br>Knowledge about illness	Lack of awarness for possible financial consequences		Promotive	Before
				Knowledge<br>Knowledge about illness	Uncertainty about length of treatment		Promotive	During

Knowledge<br>Knowledge about illness	Cancer as chronic disease	Promotive	During
Knowledge<br>Personal beliefs & attitudes	Stigma attached to financial hardship	Promotive	Before
Financial resources<br>Cancer related financial impact	Cancer related loss of income	Promotive	During
Employment<br>Pre-diagnosis work status	Paid employment at time of diagnosis	Promotive	Before
Financial resources<br>Financial support	Subsist on reduced pay or statutory sick pay for lengthy periods	Promotive	During
Employment<br>Pre-diagnosis work status	Retired	Preventive	Before
Financial resources<br>Financial commitment	Financial commitments	Promotive	Before/During
Financial resources<br>Financial commitment	Expenses for dependent children	Promotive	During
Financial resources<br>Financial support	Financial support for carers	Promotive	During
Employment<br>Direct implications	Loss of employment	Promotive	During
Financial spending<br>Direct non-medical costs	Expenses for hospital travel and parking	Promotive	During

				Financial spending<br>Direct non-medical costs	Domestic fuel bills	Promotive	During
				Financial spending<br>Direct medical costs	Prescription charges	Promotive	During
				Financial spending<br>Direct non-medical costs	telephone bills	Promotive	During
				Insurance<br>Health insurance	Increased insurance premiums	Promotive	During
				Financial spending<br>Direct non-medical costs	Healthier Food	Promotive	During
				Financial spending<br>Direct non-medical costs	Nutritional supplements	Promotive	During
				Financial spending<br>Direct non-medical costs	Complementary therapies	Promotive	During
				Financial spending<br>Direct medical costs	wigs, incontinence pads, clothes (because of weight loss/gain), post-mastectomy bras	Promotive	During
				Financial spending<br>Direct non-medical costs	Payments for cleaning, gardening and building work	Promotive	During
				Financial spending<br>Consequences of OOP	Less expenses during treatment and ealry recovery due to being debilitated	Preventive	During
Bennett (2009) [32]	Survey	The disruption of work life for a cancer patient undergoing treatment that affects current	Thematic analysis of answers to open ended questions from a survey	Employment<br>Direct implications	Loss of income	Promotive	During

		finances and future prospects for maintaining income					
				Medical (& treatment) factors<br>Side effect	Physical difficulties due to treatment effects	Promotive	During
				Employment<br>Direct implications	Sick leave	Promotive	During
				Employment<br>Employer	Supportive employers	Preventive	During
				Insurance<br>Social security	medical government benefit	Promotive	During
Fitch et al (2018) [33]	Interviews	Financial costs associated with changes and the emotional upheaval they experienced in coping with the financial burden.	Qualitative analysis of individual Interviews	Employment<br>Direct implications	Reduction of income	Promotive	During
				Financial spending<br>Direct medical costs	Additional expenses related to treatment	Promotive	During
				Employment<br>Direct implications	Patients/caregivers not able to work for a period of time	Promotive	During
				Financial resources<br>Pre-diagnosis financial status	Preexisting financial status (ref: poor)	Preventive	Before
				Insurance<br>Social security	Disability coverage (ref: no)	Preventive	Before
				Insurance<br>Health insurance	Insurance plans (ref: no)	Preventive	Before

				Financial resources<br><br>Coping	Ability to manage their finances (ref: not)	Preventive	During
				Social circumstances<br><br>Family	Having children in education	Promotive	Before
				Social circumstances<br><br>Family	Financial support of family and friends	Promotive/ Preventive	During
				Employment<br><br>Return to work	Work adjustments (work from home or part time after treatement)	Promotive	During
				Employment<br><br>Employer	Loss of access to benefit plans	Promotive	During
				Employment<br><br>Employer	income coverage from employee benefit plans	Preventive	Before
				Employment<br><br>Pre-diagnosis work status	Being self-employed	Promotive	Before
				Financial resources<br><br>Coping	Using savings	Promotive	During
Fitch et al (2020) [34]	Survey	Cancer patient perspectives regarding the impact of financial burden on their daily lives or the kinds of choices individuals make to cope with the situation over time. If their coping mechanisms fail, individuals may experience heightened financial distress.	Qualitative analysis of asnwers to open ended questions from a survey	Financial spending<br><br>Cancer-related total costs	Additional expenses	Promotive	During
				Financial spending<br><br>Consequences of OOP	Able to manage through various means to cover expenditures	Preventive	During

	Insurance<br>Health insurance	Time between travel expenses and reimbursement	Promotive	During
	Financial resources<br>Financial commitment	Mortgage	Promotive	Before
	Employment<br>Direct implications	Redued income	Promotive	During
	Employment<br>Direct implications	Changes in work situation	Promotive	During
	Financial resources<br>Cancer related financial impact	Reduced reserves	Promotive	During
	Employment<br>Direct implications	Due to side effects not able to work	Promotive	During
	Financial resources<br>Pre-diagnosis financial status	Financial status at the diagnosis	Promotive	Before
	Social circumstances<br>Family	Both partners ill at the same time	Promotive	During
	Employment<br>Direct implications	No one able to work	Promotive	During
	Socio-demographic factors<br>Familial status	Having children	Promotive	Before
	Insurance<br>Health insurance	Private health insurance	Preventive	Before

				Employment<br>Employer	Employers benefit plan	Preventive	Before
				Social circumstances<br>Support	Support of family and friends	Preventive	During
				Employment<br>Employer	Caregivers' work benefit plans	Preventive	Before
Gray et al (2004) [35]	Focus groups	Concerns about finance	Qualitative analysis of focus groups	Socio-demographic factors<br>Geography	Living in rural areas	Promotive	Before
				Employment<br>Pre-diagnosis work status	Unemployment	Promotive	During
				Insurance<br>Health insurance	Limited assistence	Promotive	During
				Socio-demographic factors<br>Geography	Treatment in urban centres	Promotive	During
				Financial spending<br>Direct non-medical costs	Phone calls	Promotive	During
				Medical (& treatment) factors<br>Side effect	Side effect medication	Promotive	During
				Financial spending<br>Direct medical costs	Prosthetics	Promotive	During
				Socio-demographic factors<br>Familial status	Having children	Promotive	Before

Grinyer (2007) [36]	Interviews	Not stated	Qualitative analysis of individual Interviews	Social circumstances<br>Support	Returning to family home	Promotive/ Preventive	During
				Socio-demographic factors<br>Age	Age	Promotive	Before
				Insurance<br>Social security	Benefit system for younger people	Promotive	Before
				Insurance<br>Health insurance	Lack of paid-up contributions	Promotive	Before
				Knowledge<br>Access to social benefits	Lack of knowledge and confidence in dealing with a bureaucratic system	Promotive	Before
				Knowledge<br>Getting assistance	Assistance by social worker	Preventive	During
Kane et al (2020) [37]	Interviews	Not stated	Explorative qualitative analysis of individual Interviews	Employment<br>Direct implications	Loss of income	Promotive	During
				Social circumstances<br>Family	Having a family	Promotive	Before
				Financial spending<br>Direct non-medical costs	Transportation expenses	Promotive	During
				Financial spending<br>Direct medical costs	Expenses for certain medications	Promotive	During
				Financial spending<br>Direct non-medical costs	Expenses for childcare	Promotive	During

				Financial spending<br>Direct non-medical costs	Expenses for home care	Promotive	During
				Financial spending<br>Direct non-medical costs	Expenses for nutritional or food supplements	Promotive	During
				Financial spending<br>Direct medical costs	Expenses for medical supplies	Promotive	During
				Insurance<br>Social security	Disability or health benefits	Preventive	Before
McGrath et al (1999) [38]	Interviews	Significant worsening of financial position	Qualitative analysis of individual Interviews	Employment<br>Direct implications	Unable to resume their work	Promotive	During
				Socio-demographic factors<br>Geography	Living in rural areas	Promotive	Before
				Financial spending<br>Direct medical costs	Additional costs for medical bills	Promotive	During
				Financial spending<br>Direct non-medical costs	Fares for travel	Promotive	During
				Financial spending<br>Direct non-medical costs	Incidental expenses	Promotive	During
				Financial spending<br>Direct non-medical costs	Cost involved in making arrangements for absence during treatment	Promotive	During
				Financial spending<br>Direct medical costs	Paying for tests	Promotive	During

				Financial spending<br>Direct non-medical costs	Covering alternative therapies	Promotive	During
				Financial spending<br>Direct medical costs	Wigs and protheses	Promotive	During
				Financial spending<br>Direct medical costs	Costs for doctors in the rural areas	Promotive	Before
				Financial spending<br>Direct non-medical costs	Costs of STD telephone calls	Promotive	During
				Financial spending<br>Direct non-medical costs	Travel to medical treatment	Promotive	During
				Social circumstances<br>Financial role	Major financially breadwinner of family	Promotive	Before
				Employment<br>Pre-diagnosis work status	Self-employed	Promotive	Before
				Financial resources<br>Financial commitment	Having property	Promotive	Before
McGrath (2000) [39]	Interviews	financial impact on families	Qualitative analysis of individual Interviews with patients and their families	Financial spending<br>Direct non-medical costs	Increased cost of shopping	Promotive	During
				Financial spending<br>Direct non-medical costs	Telephone calls	Promotive	During

Social circumstances<br>Support	Loss of support of local voluntary organisation	Promotive	During
Financial spending<br>Direct medical costs	Addiditional medical costs	Promotive	During
Financial spending<br>Direct non-medical costs	Accommodation costs	Promotive	During
Financial spending<br>Direct non-medical costs	Cost of maintaining home in absence after pets etc in caretakers absence	Promotive	During
Financial spending<br>Direct non-medical costs	Incidental costs	Promotive	During
Medical (& treatment) factors<br>Treatment	(Free) Accommodation at Leukaemia Foundation of Queensland's Centres	Preventive	During
Employment<br>Employer	Employer support	Preventive	During
Social circumstances<br>Financial role	"Bread winner" not affected	Preventive	Before
Financial resources<br>Coping	Credit Cards	Preventive	During
Insurance<br>Health insurance	Prescribed drugs of PBS	Preventive	During
Employment<br>Pre-diagnosis work status	Part-time income	Preventive	During

				Financial resources<br>Pre-diagnosis financial status	‘Nest-egg’ available	Preventive	Before
				Financial resources<br>Financial commitment	Home owner	Preventive	Before
				Financial resources<br>Financial support	Income insurance	Preventive	Before
				Financial resources<br>Financial commitment	Threat of selling family home	Promotive	During
				Financial resources<br>Financial commitment	Home and car re-payments	Promotive	During
				Financial resources<br>Cancer related financial impact	Erode ´buffer zone´	Promotive	During
				Financial resources<br>Pre-diagnosis financial status	No savings to start with	Promotive	Before
				Financial resources<br>Financial support	Long-term on social security benefits	Promotive	During
				Financial resources<br>Cancer related financial impact	Credit cards	Promotive	During
				Employment<br>Pre-diagnosis work status	Self-employed	Promotive	Before
Moffatt et al (2010) [40]	Interviews	Dfficulties affording the additional resources required to cope with illness	Qualitative analysis of individual Interviews	Financial resources<br>Pre-diagnosis financial status	Working partner earns a high income	Preventive	Before

		Insurance<br>Health insurance	Well covered by private health insurance	Preventive	Before
		Financial resources<br>Financial support	Well covered by mortgage protection	Preventive	Before
		Employment<br>Pre-diagnosis work status	Better payed job	Preventive	Before
		Employment<br>Employer	Working in the public sector	Preventive	Before
		Employment<br>Employer	Not qualifying for occupational sick benefits	Promotive	Before
		Insurance<br>Social security	Relying on Statutory Sick Pay or Employment Support Allowance	Promotive	Before
		Employment<br>Direct implications	Considerable drop in household income	Promotive	During
		Financial spending<br>Cancer-related total costs	Significant additional expenditure	Promotive	During
		Knowledge<br>Getting assistance	Assistance from welfare rights advisors	Preventive	During
		Social circumstances<br>Family	Having young children at home	Promotive	Before
		Social circumstances<br>Financial role	Being the ´main´ earner	Promotive	Before

Employment<br>Pre-diagnosis work status	Self-employed without illness insurance	Promotive	Before
Financial spending<br>Direct non-medical costs	Petrol and parking costs for hospital visits	Promotive	During
Financial spending<br>Direct non-medical costs	Addional heating costs	Promotive	During
Financial spending<br>Direct non-medical costs	Dietary needs	Promotive	During
Financial spending<br>Direct non-medical costs	Clothing needs	Promotive	During
Financial spending<br>Consequences of OOP	Buying cheaper brands of food	Promotive	During
Financial spending<br>Consequences of OOP	Economising on other household bills	Promotive	During
Social circumstances<br>Social activities	Reducing social activities	Promotive	During
Social circumstances<br>Social activities	Reducing leisure activities	Promotive	During
Social circumstances<br>Support	Borrowing cash from other family members	Preventive	During
Social circumstances<br>Social activities	Continuing with activities such as sports and music that usally involved some expense (trying to maintain a sense of normality and routine for children)	Promotive	During

				Knowledge<br>Access to social benefits	Lack of knowledge about available benefits	Promotive	During
				Knowledge<br>Getting assistance	Help from welfare rights advisers	Preventive	During
				Knowledge<br>Personal beliefs & attitudes	Attitudinal factors that influenced their propensity to claim (e.g. values of hard work, 'making do' and self reliance)	Promotive	Before
				Knowledge<br>Personal beliefs & attitudes	Beliefs about the extent and severity of illness	Promotive	During
				Knowledge<br>Getting assistance	Time between diagnosis and receipt of advice	Promotive	During
Moffatt et al (2012) [41]	Interviews	The financial difficulties experienced due to a temporary or permanent loss of earnings as well as the additional costs associated with cancer	Qualitative analysis of individual Interviews	Employment<br>Direct implications	Lost earnings as a result of temporary or permanent cessation of work	Promotive	During
				Knowledge<br>Getting assistance	Assistance applying for and receive illness and incapacity benefits	Promotive	During
				Financial spending<br>Direct non-medical costs	Travel and parking	Promotive	During
				Financial spending<br>Direct non-medical costs	Dietary requirements	Promotive	During
				Financial spending<br>Direct non-medical costs	Heating and clothing costs	Promotive	During

				Financial spending<br>Direct non-medical costs	Adaptations to the home	Promotive	During
				Financial spending<br>Direct non-medical costs	Paying for extra help	Promotive	During
				Knowledge<br>Access to social benefits	Lack of knowledge about available benefits and eligibility criteria	Promotive	During
				Knowledge<br>Personal beliefs & attitudes	Negative attitudes towards claiming benefits	Promotive	Before
				Knowledge<br>Personal beliefs & attitudes	Unpleasant dealings with the benefits system in the past	Promotive	Before
				Knowledge<br>Getting assistance	Professionals who did not alert patients to benefit entitlements	Promotive	During
				Knowledge<br>Access to social benefits	Benefits system is time consuming and complex	Promotive	Before
				Medical (& treatment) factors<br>Side effect	Participant accounts of fatigue, pain, nausea or inability to concentrate deterred individuals from the lengthy and complex process of establishing eligibility criteria and completing lengthy benefit applications without assistance	Promotive	During
				Knowledge<br>Getting assistance	Time between diagnosis and receipt of advice	Promotive	During
Moffatt et al (2015) [42]	Interviews	The financial difficulties experienced due to a temporary or permanent loss of earnings as well as the additional costs associated with cancer	Qualitative longitudinal analysis of individual Interviews	Employment<br>Direct implications	Drop in income	Promotive	During

Employment<br>Pre-diagnosis work status	Type of job	Promotive/ Preventive	Before
Employment<br>Employer	Eligibility for occupational sickness protection	Preventive	Before
Employment<br>Pre-diagnosis work status	High paid job	Preventive	Before
Employment<br>Pre-diagnosis work status	White-collar public sector employment	Preventive	Before
Financial resources<br>Cancer related financial impact	Affect of ability to meet bills, housing payments and other bills	Promotive	During
Financial spending<br>Consequences of OOP	Economising on household expenditure	Promotive	During
Financial resources<br>Coping	Borrowing cash	Promotive	During
Social circumstances<br>Support	Relying on others for transport to hospital	Promotive	During
Financial resources<br>Coping	Using up savings	Promotive	During
Financial resources<br>Coping	Accuring debt	Promotive	During
Social circumstances<br>Family	Carers/partners reducing working hours to support and spend time with patients (further reducing household income)	Promotive	During

				Social circumstances<br>Support	Carers/partners reluctantly increasing their [work] hours in an attempt to offset lost income	Promotive	During
				Employment<br>Direct implications	Cancer related financial impact of treatment on ability to work	Promotive/ Preventive	During
				Employment<br>Pre-diagnosis work status	Self-employment	Promotive	Before
Schröder et al (2020) [43]	Interviews	The social impact of cancer, particu- larly on employment and its financial consequences	Qualitative analysis of semi-structured interviews	Financial spending<br>Consequences of OOP	Accommodation (delaying rent or postponing maintenance of a residential property)	Promotive	Before
				Financial spending<br>Consequences of OOP	Food (buying food on sale or cooking inexpensive dishes)	Promotive	During
				Financial spending<br>Consequences of OOP	Utility costs (reducing energy, water consumption)	Promotive	During
				Financial spending<br>Consequences of OOP	Transportation (postponing maintenance of a car, selling a car, or foregoing purchasing a new car)	Promotive	During
				Financial spending<br>Consequences of OOP	Clothing (being unable to buy new clothes for children or buying second-hand clothes)	Promotive	During
				Financial spending<br>Consequences of OOP	Delaying major purchases	Promotive	During
				Financial spending<br>Consequences of OOP	Holidays (forego or choose less expensive options)	Promotive	During
				Financial spending<br>Consequences of OOP	Reducing hobbies, social contacts, and leisure activities	Promotive	During

Financial spending<br>Consequences of OOP	Preventive new purchases	Promotive	During
Financial spending<br>Consequences of OOP	Forego transport service due to high OOP payments	Promotive	During
Financial spending<br>Consequences of OOP	Forego medical remedies due to high OOP payments	Promotive	During
Insurance<br>Health insurance	Private health insurance	Promotive	During
Financial resources<br>Cancer related financial impact	General	Promotive	During
Financial resources<br>Coping	Pension savings	Promotive	During
Financial resources<br>Coping	Using savings	Promotive	During
Employment<br>Return to work	Returned to work quickly	Promotive	During
Employment<br>Return to work	Marginal employment	Promotive	During
Social circumstances<br>Support	Family and friends	Promotive	During
Insurance<br>Social security	Institutional support	Promotive	During

				Financial resources<br>Coping	Incurring bank debts	Promotive	During
				Medical (& treatment) factors<br>Treatment	Making health-related decisions	Promotive	During
Slavova-Azmanova et al (2020) [44]	Survey	Costs associated with cancer treatment and how they affected ones life	Qualitative analysis of answers to open-ended questions	Insurance<br>Health insurance		Promotive/ Preventive	During
				Employment<br>Pre-diagnosis work status	Pensioners	Promotive/ Preventive	Before
				Employment<br>Employer	Leave available	Preventive	Before
				Employment<br>Direct implications	Consequences on participants' employment and ability to work	Promotive	During
				Employment<br>Direct implications	Consequences on partner's income and employment	Promotive	During
				Financial resources<br>Coping	Savings, superannuation, credit cards, borrow from family	Promotive	During
				Financial resources<br>Financial support	Insurances - trauma, life, income protection	Preventive	Before
				Financial resources<br>Financial support	CentreLink assistance	Promotive	During
				Insurance<br>Health insurance	PATS	Promotive/ Preventive	During

				Insurance<br>Health insurance	Concession cards	Preventive	Before
				Social circumstances<br>Support	Community and NGOs' support	Preventive	During
				Social circumstances<br>Support	Family support	Promotive/ Preventive	During
				Knowledge<br>Access to healthcare	Unknown expenses, not knowing where treatment may be available and waiting times	Promotive	During
				Knowledge<br>Access to healthcare	Looking up for different providers	Promotive	During
				Knowledge<br>Access to healthcare	Knowing how to navigate the health care system	Promotive	Before
Timmons et al (2013a) [45]	Interviews	All facets of burden	Qualitative analysis of individual interviews	Financial spending<br>Direct medical costs	Hospital bills	Promotive	During
				Financial spending<br>Direct medical costs	Consultant visits	Promotive	During
				Financial spending<br>Direct medical costs	Primay care physician visits	Promotive	During
				Financial spending<br>Direct medical costs	Procedures	Promotive	During
				Financial spending<br>Direct medical costs	Prescription medications	Promotive	During

	Financial spending<br>Direct medical costs	Convalescence after surgery	Promotive	During
	Financial spending<br>Direct medical costs	Counselling	Promotive	During
	Financial spending<br>Direct medical costs	Manual lymph drainage	Promotive	During
	Financial spending<br>Direct medical costs	Wigs/bandanas/night caps	Promotive	During
	Financial spending<br>Direct medical costs	Prothesis bras/related items	Promotive	During
	Financial spending<br>Direct medical costs	Over-the-counter medications	Promotive	During
	Financial spending<br>Direct non-medical costs	New clothes/second-hand clothes	Promotive	During
	Financial spending<br>Direct non-medical costs	Transport	Promotive	During
	Financial spending<br>Direct non-medical costs	Hospital parking	Promotive	During
	Financial spending<br>Direct non-medical costs	Meals (when attending hospital)	Promotive	During
	Financial spending<br>Direct non-medical costs	Overnight accomodation (when having treatment)	Promotive	During

	Financial spending<br>Direct non-medical costs	Childcare costs (babysitting or crèche; when attending hospital)	Promotive	During
	Financial spending<br>Direct non-medical costs	Heating bills	Promotive	During
	Financial spending<br>Direct non-medical costs	Telephone bills	Promotive	During
	Financial spending<br>Direct non-medical costs	Homecare	Promotive	During
	Financial spending<br>Direct non-medical costs	Healthy eating	Promotive	During
	Financial spending<br>Direct non-medical costs	House modifications	Promotive	During
	Employment<br>Direct implications	Workplace participation post-diagnosis	Promotive/<br>Preventive	During
	Employment<br>Direct implications	Factors influencing whether cancer patients work during treatment	Promotive/<br>Preventive	During
	Employment<br>Direct implications	Cancer related financial impact of cancer on employment of other family members	Promotive/<br>Preventive	During
	Employment<br>Direct implications	Factors influencing time-off work for other family members	Promotive/<br>Preventive	During
	Employment<br>Direct implications	Factors influencing change in income	Promotive/<br>Preventive	During

				Employment<br>Direct implications	Cancer related financial impact of change in income	Promotive/ Preventive	During
				Employment<br>Return to work	Factors influencing return to work	Promotive/ Preventive	After
Timmons et al (2013b) [46]	Interview	Experiencing "extra costs" or "financial difficulties" because of cancer	Qualitative analysis of individual interviews	Employment<br>Pre-diagnosis work status	Working at diagnosis	Promotive	Before
				Socio-demographic factors<br>Familial status	Having young children	Promotive	Before
				Socio-demographic factors<br>Familial status	Being a lone parent	Promotive	Before
				Socio-demographic factors<br>Age	Being younger	Promotive	Before
				Insurance<br>Health insurance	Not having a medical card	Promotive	Before
				Financial resources<br>Pre-diagnosis financial status	Low income pre-diagnosis	Promotive	Before
				Financial resources<br>Financial support	Receiving social welfare benefits	Promotive	Before
				Financial resources<br>Pre-diagnosis financial status	Having no/few savings	Promotive	Before

	Financial resources<br>Financial commitment	Having a mortgage	Promotive	Before
	Socio-demographic factors<br>Geography	Living in remote/rural areas	Promotive	Before
	Insurance<br>Social security	Ineligible for social welfare benefits (e.g. self-employed, foreign nationals, farmers)	Promotive	Before
	Socio-demographic factors<br>Familial status	Living alone	Promotive	Before
	Socio-demographic factors<br>Age	Being elderly	Promotive	Before
	Social circumstances<br>Family	Socially isolated, lack of family/social support	Promotive	During
	Medical (& treatment) factors<br>Treatment	Receiving inpatient treatment	Preventive	During
	Medical (& treatment) factors<br>Treatment	Access to hospital accommodation (e.g. during radiotherapy)	Preventive	During
	Social circumstances<br>Support	Supportive family and friends able to provide financial or practical help	Preventive	During
	Financial resources<br>Financial support	Receiving full sick pay	Preventive	During
	Financial resources<br>Pre-diagnosis financial status	Having other financial resources (e.g. savings, private health insurance, illness insurance)	Preventive	Before

References
1. Akechi, T.; Okuyama, T.; Uchida, M.; Nakaguchi, T.; Ito, Y.; Yamashita, H.; Toyama, T.; Komatsu, H.; Kizawa, Y.; Wada, M. Perceived needs, psychological distress and quality of life of elderly cancer patients. *Jpn. J. Clin. Oncol.* **2012**, *42*, 704–710, doi:10.1093/jjco/hys075.
2. Arndt, V.; Merx, H.; Stegmaier, C.; Ziegler, H.; Brenner, H. Quality of life in patients with colorectal cancer 1 year after diagnosis compared with the general population: a population-based study. *J. Clin. Oncol.* **2004**, *22*, 4829–4836, doi:10.1200/JCO.2004.02.018.
3. Arndt, V.; Koch-Gallenkamp, L.; Jansen, L.; Bertram, H.; Eberle, A.; Holleczek, B.; Schmid-Höpfner, S.; Waldmann, A.; Zeissig, S.R.; Brenner, H. Quality of life in long-term and very long-term cancer survivors versus population controls in Germany. *Acta Oncol.* **2017**, *56*, 190–197, doi:10.1080/0284186X.2016.1266089.
4. Arndt, V.; Koch-Gallenkamp, L.; Bertram, H.; Eberle, A.; Holleczek, B.; Pritzkuleit, R.; Waldeyer-Sauerland, M.; Waldmann, A.; Zeissig, S.R.; Doege, D.; et al. Return to work after cancer. A multi-regional population-based study from Germany. *Acta Oncol.* **2019**, *58*, 811–818, doi:10.1080/0284186X.2018.1557341.
5. Barbaret, C.; Brosse, C.; Rhondali, W.; Ruer, M.; Monsarrat, L.; Michaud, P.; Schott, A.M.; Delgado-Guay, M.; Bruera, E.; Sanchez, S.; et al. Financial distress in patients with advanced cancer. *PLoS One* **2017**, *12*, e0176470, doi:10.1371/journal.pone.0176470.
6. Barbaret, C.; Delgado-Guay, M.O.; Sanchez, S.; Brosse, C.; Ruer, M.; Rhondali, W.; Monsarrat, L.; Michaud, P.; Schott, A.M.; Bruera, E.; et al. Inequalities in Financial Distress, Symptoms, and Quality of Life Among Patients with Advanced Cancer in France and the U.S. *Oncologist* **2019**, *24*, 1121–1127, doi:10.1634/theoncologist.2018-0353.
7. Büttner, M.; König, H.-H.; Löbner, M.; Briest, S.; Konnopka, A.; Dietz, A.; Riedel-Heller, S.; Singer, S. Out-of-pocket-payments and the financial burden of 502 cancer patients of working age in Germany: results from a longitudinal study. *Support. Care Cancer* **2019**, *27*, 2221–2228, doi:10.1007/s00520-018-4498-1.
8. Doege, D.; Thong, M.S.-Y.; Koch-Gallenkamp, L.; Bertram, H.; Eberle, A.; Holleczek, B.; Pritzkuleit, R.; Waldeyer-Sauerland, M.; Waldmann, A.; Zeissig, S.R.; et al. Health-related quality of life in long-term disease-free breast cancer survivors versus female population controls in Germany. *Breast Cancer Res. Treat.* **2019**, *175*, 499–510, doi:10.1007/s10549-019-05188-x.
9. Egestad, H.; Nieder, C. Undesirable financial effects of head and neck cancer radiotherapy during the initial treatment period. *Int. J. Circumpolar Health* **2015**, *74*, 26686, doi:10.3402/ijch.v74.26686.
10. Ezeife, D.A.; Morganstein, B.J.; Lau, S.; Law, J.H.; Le, L.W.; Bredle, J.; Cella, D.; Doherty, M.K.; Bradbury, P.; Liu, G.; et al. Financial Burden Among Patients With Lung Cancer in a Publically Funded Health Care System. *Clin. Lung Cancer* **2019**, *20*, 231–236, doi:10.1016/j.cllc.2018.12.010.
11. Gordon, L.G.; Walker, S.M.; Mervin, M.C.; Lowe, A.; Smith, D.P.; Gardiner, R.A.; Chambers, S.K. Financial toxicity: a potential side effect of prostate cancer treatment among Australian men. *Eur. J. Cancer Care (Engl)* **2017**, *26*, doi:10.1111/ecc.12392.
12. Gordon, L.G.; Beesley, V.L.; Mihala, G.; Koczwara, B.; Lynch, B.M. Reduced employment and financial hardship among middle-aged individuals with colorectal cancer. *Eur. J. Cancer Care (Engl)* **2017**, *26*, doi:10.1111/ecc.12744.
13. Gordon, L.G.; Elliott, T.M.; Wakelin, K.; Leyden, S.; Leyden, J.; Michael, M.; Pavlakis, N.; Mumford, J.; Segelov, E.; Wyld, D.K. The Economic Impact on Australian Patients with Neuroendocrine Tumours. *Patient* **2020**, *13*, 363–373, doi:10.1007/s40271-020-00412-z.
14. Hanly, P.; Maguire, R.; Ceilleachair, A.O.; Sharp, L. Financial hardship associated with colorectal cancer survivorship: The role of asset depletion and debt accumulation. *Psychooncology.* **2018**, *27*, 2165–2171, doi:10.1002/pon.4786.
15. Honda, K.; Gyawali, B.; Ando, M.; Kumanishi, R.; Kato, K.; Sugiyama, K.; Mitani, S.; Masuishi, T.; Narita, Y.; Bando, H.; et al. Prospective Survey of Financial Toxicity Measured by the Comprehensive Score for Financial Toxicity in Japanese Patients With Cancer. *J. Glob. Oncol.* **2019**, *5*, 1–8, doi:10.1200/JGO.19.00003.
16. Ivanauskienė, R.; Padaiga, Ž.; Šimoliūnienė, R.; Smailytė, G.; Domeikienė, A. Well-being of newly diagnosed women with breast cancer: which factors matter more? *Support. Care Cancer* **2014**, *22*, 519–526, doi:10.1007/s00520-013-2005-2.
17. Kaptein, A.A.; Yamaoka, K.; Snoei, L.; van der Kloot, W.A.; Inoue, K.; Tabei, T.; Kroep, J.R.; Krol-Warmerdam, E.; Ranke, G.; Meirink, C.; et al. Illness perceptions and quality of life in Japanese and Dutch women with breast cancer. *J. Psychosoc. Oncol.* **2013**, *31*, 83–102, doi:10.1080/07347332.2012.741092.
18. Koch, L.; Jansen, L.; Herrmann, A.; Stegmaier, C.; Holleczek, B.; Singer, S.; Brenner, H.; Arndt, V. Quality of life in long-term breast cancer survivors - a 10-year longitudinal population-based study. *Acta Oncol.* **2013**, *52*, 1119–1128, doi:10.3109/0284186X.2013.774461.
19. Koskinen, J.-P.; Färkkilä, N.; Sintonen, H.; Saarto, T.; Taari, K.; Roine, R.P. The association of financial difficulties and out-of-pocket payments with health-related quality of life among breast, prostate and colorectal cancer patients. *Acta Oncol.* **2019**, *58*, 1062–1068, doi:10.1080/0284186X.2019.1592218.
20. Lu, L.; O'Sullivan, E.; Sharp, L. Cancer-related financial hardship among head and neck cancer survivors: Risk factors and associations with health-related quality of life. *Psychooncology.* **2019**, *28*, 863–871, doi:10.1002/pon.5034.
21. Mandaliya, H.; Ansari, Z.; Evans, T.; Oldmeadow, C.; George, M. Psychosocial Analysis of Cancer Survivors in Rural Australia: Focus on Demographics, Quality of Life and Financial Domains. *Asian Pac. J. Cancer Prev.* **2016**, *17*, 2459–2464.
22. McLean, L.; Hong, W.; McLachlan, S.-A. Financial toxicity in patients with cancer attending a public Australian tertiary hospital: A pilot study. *Asia Pac. J. Clin. Oncol.* **2020**, doi:10.1111/ajco.13448.
23. McNeil, R.J.; McCarthy, M.; Dunt, D.; Thompson, K.; Kosola, S.; Orme, L.; Drew, S.; Sawyer, S. Financial Challenges of Cancer for Adolescents and Young Adults and Their Parent Caregivers. *Social Work Research* **2019**, *43*, 17–30, doi:10.1093/swr/svy027.

24. Mehlis, K.; Witte, J.; Surmann, B.; Kudlich, M.; Apostolidis, L.; Walther, J.; Jäger, D.; Greiner, W.; Winkler, E.C. The patient-level effect of the cost of Cancer care - financial burden in German Cancer patients. *BMC Cancer* **2020**, *20*, 529, doi:10.1186/s12885-020-07028-4.
25. Mercadante, S.; Aielli, F.; Adile, C.; Bonanno, G.; Casuccio, A. Financial distress and its impact on symptom expression in advanced cancer patients. *Support. Care Cancer* **2021**, *29*, 485–490, doi:10.1007/s00520-020-05507-9.
26. Paul, C.L.; Hall, A.E.; Carey, M.L.; Cameron, E.C.; Clinton-McHarg, T. Access to care and impacts of cancer on daily life: do they differ for metropolitan versus regional hematological cancer survivors? *J. Rural Health* **2013**, *29 Suppl 1*, s43-50, doi:10.1111/jrh.12020.
27. Pearce, A.; Tomalin, B.; Kaambwa, B.; Horevoorts, N.; Duijts, S.; Mols, F.; van de Poll-Franse, L.; Koczwara, B. Financial toxicity is more than costs of care: the relationship between employment and financial toxicity in long-term cancer survivors. *J. Cancer Surviv.* **2019**, *13*, 10–20, doi:10.1007/s11764-018-0723-7.
28. Schmidt, M.E.; Scherer, S.; Wiskemann, J.; Steindorf, K. Return to work after breast cancer: The role of treatment-related side effects and potential impact on quality of life. *Eur. J. Cancer Care (Engl)* **2019**, *28*, e13051, doi:10.1111/ecc.13051.
29. Sharp, L.; Timmons, A. Pre-diagnosis employment status and financial circumstances predict cancer-related financial stress and strain among breast and prostate cancer survivors. *Support. Care Cancer* **2016**, *24*, 699–709, doi:10.1007/s00520-015-2832-4.
30. Zucca, A.; Boyes, A.; Newling, G.; Hall, A.; Girgis, A. Travelling all over the countryside: travel-related burden and financial difficulties reported by cancer patients in New South Wales and Victoria. *Aust. J. Rural Health* **2011**, *19*, 298–305, doi:10.1111/j.1440-1584.2011.01232.x.
31. Amir, Z.; Wilson, K.; Hennings, J.; Young, A. The meaning of cancer: implications for family finances and consequent impact on lifestyle, activities, roles and relationships. *Psychooncology.* **2012**, *21*, 1167–1174, doi:10.1002/pon.2021.
32. Bennett, J.A.; Brown, P.; Cameron, L.; Whitehead, L.C.; Porter, D.; McPherson, K.M. Changes in employment and household income during the 24 months following a cancer diagnosis. *Support. Care Cancer* **2009**, *17*, 1057–1064, doi:10.1007/s00520-008-0540-z.
33. Fitch, M.; Longo, C.J. Exploring the impact of out-of-pocket costs on the quality of life of Canadian cancer patients. *J. Psychosoc. Oncol.* **2018**, *36*, 582–596, doi:10.1080/07347332.2018.1486937.
34. Fitch, M.I.; Longo, C.J.; Chan, R.J. Cancer patients' perspectives on financial burden in a universal healthcare system: Analysis of qualitative data from participants from 20 provincial cancer centers in Canada. *Patient Educ. Couns.* **2021**, *104*, 903–910, doi:10.1016/j.pec.2020.08.013.
35. Gray, R.E.; James, P.; Manthorne, J.; Gould, J.; Fitch, M.I. A consultation with Canadian rural women with breast cancer. *Health Expect.* **2004**, *7*, 40–50, doi:10.1046/j.1369-6513.2003.00248.x.
36. Grinyer, A. The biographical impact of teenage and adolescent cancer. *Chronic Illn.* **2007**, *3*, 265–277, doi:10.1177/1742395307085335.
37. Kane, D.; Rajacich, D.; Andary, C. Experiences of cancer patients' return to work. *Can. Oncol. Nurs. J.* **2020**, *30*, 113–118, doi:10.5737/23688076302113118.
38. McGrath, P.; Patterson, C.; Yates, P.; Treloar, S.; Oldenburg, B.; Loos, C. A study of postdiagnosis breast cancer concerns for women living in rural and remote Queensland. Part II: Support issues. *Aust. J. Rural Health* **1999**, *7*, 43–52, doi:10.1046/j.1440-1584.1999.00216.x.
39. McGrath, P. "It's horrendous--but really, what can you do?" Preliminary findings on financial impact of relocation for specialist treatment. *Aust. Health Rev.* **2000**, *23*, 94–103, doi:10.1071/ah000094.
40. Moffatt, S.; Noble, E.; Exley, C. "Done more for me in a fortnight than anybody done in all me life." How welfare rights advice can help people with cancer. *BMC Health Serv. Res.* **2010**, *10*, 259, doi:10.1186/1472-6963-10-259.
41. Moffatt, S.; Noble, E.; White, M. Addressing the financial consequences of cancer: qualitative evaluation of a welfare rights advice service. *PLoS One* **2012**, *7*, e42979, doi:10.1371/journal.pone.0042979.
42. Moffatt, S.; Noble, E. Work or welfare after cancer? Explorations of identity and stigma. *Sociol. Health Illn.* **2015**, *37*, 1191–1205, doi:10.1111/1467-9566.12303.
43. Schröder, S.L.; Schumann, N.; Fink, A.; Richter, M. Coping mechanisms for financial toxicity: a qualitative study of cancer patients' experiences in Germany. *Support. Care Cancer* **2020**, *28*, 1131–1139, doi:10.1007/s00520-019-04915-w.
44. Slavova-Azmanova, N.S.; Newton, J.C.; Saunders, C.; Johnson, C.E. 'Biggest factors in having cancer were costs and no entitlement to compensation'-The determinants of out-of-pocket costs for cancer care through the lenses of rural and outer metropolitan Western Australians. *Aust. J. Rural Health* **2020**, *28*, 588–602, doi:10.1111/ajr.12686.
45. Timmons, A.; Gooberman-Hill, R.; Sharp, L. "It's at a time in your life when you are most vulnerable": a qualitative exploration of the financial impact of a cancer diagnosis and implications for financial protection in health. *PLoS One* **2013**, *8*, e77549, doi:10.1371/journal.pone.0077549.
46. Timmons, A.; Gooberman-Hill, R.; Sharp, L. The multidimensional nature of the financial and economic burden of a cancer diagnosis on patients and their families: qualitative findings from a country with a mixed public-private healthcare system. *Support. Care Cancer* **2013**, *21*, 107–117, doi:10.1007/s00520-012-1498-4.
